# A phenol-enriched cuticle is ancestral to lignin evolution in land plants

**DOI:** 10.1038/ncomms14713

**Published:** 2017-03-08

**Authors:** Hugues Renault, Annette Alber, Nelly A. Horst, Alexandra Basilio Lopes, Eric A. Fich, Lucie Kriegshauser, Gertrud Wiedemann, Pascaline Ullmann, Laurence Herrgott, Mathieu Erhardt, Emmanuelle Pineau, Jürgen Ehlting, Martine Schmitt, Jocelyn K. C. Rose, Ralf Reski, Danièle Werck-Reichhart

**Affiliations:** 1 University of Strasbourg, Institute of Plant Molecular Biology, Centre National de la Recherche Scientifique, 12 rue du Général Zimmer, 67000 Strasbourg, France; 2 Faculty of Biology, Chair of Plant Biotechnology, University of Freiburg, Schaenzlestrasse 1, 79104 Freiburg, Germany; 3 University of Strasbourg Institute for Advanced Study, 5 allée du Général Rouvillois, 67000 Strasbourg, France; 4 Freiburg Institute for Advanced Studies, University of Freiburg, Albertstraße 19, 79104 Freiburg, Germany; 5 Department of Biology & Centre for Forest Biology, University of Victoria, British Columbia, Canada V8P 5C2; 6 Laboratoire d'Innovation Thérapeutique, UMR CNRS 7200, Université de Strasbourg, 74 route du Rhin, 67401 Illkirch, France; 7 Plant Biology Section, School of Integrative Plant Science, Cornell University, Ithaca, New York 14853, USA; 8 BIOSS - Centre for Biological Signalling Studies, 79104 Freiburg, Germany

## Abstract

Lignin, one of the most abundant biopolymers on Earth, derives from the plant phenolic metabolism. It appeared upon terrestrialization and is thought critical for plant colonization of land. Early diverging land plants do not form lignin, but already have elements of its biosynthetic machinery. Here we delete in a moss the P450 oxygenase that defines the entry point in angiosperm lignin metabolism, and find that its pre-lignin pathway is essential for development. This pathway does not involve biochemical regulation via shikimate coupling, but instead is coupled with ascorbate catabolism, and controls the synthesis of the moss cuticle, which prevents desiccation and organ fusion. These cuticles share common features with lignin, cutin and suberin, and may represent the extant representative of a common ancestor. Our results demonstrate a critical role for the ancestral phenolic metabolism in moss erect growth and cuticle permeability, consistent with importance in plant adaptation to terrestrial conditions.

## Abstract (web summary)

The phenolic polymer lignin is thought to have contributed to adaptation of early land plants to terrestrial environments. Here Renault *et al*. show that moss, which does not produce lignin, contains an ancestral phenolic metabolism pathway that produces a phenol-enriched cuticle and prevents desiccation.

Land plants evolved from charophycean freshwater green algae around 450 Ma (refs 1, 2). Key challenges associated with terrestrialization would have included increased biomechanical stresses, desiccation, rapid temperature fluctuations and higher light intensity, particularly in the ultraviolet range. Colonization of land would therefore have required major metabolic adaptations in the form of ultraviolet screens, antioxidants and precursors for structural biopolymers to resist desiccation but to allow gas exchange between the plant and the environment to ensure proper photosynthesis. Three types of complex hydrophobic extracellular biopolymers are generally described as contributing to permeability control and water transport in the vegetative tissues of vascular plants, the evolution of which was likely guided by similar selective pressures: cutin, a lipid-derived component of the cuticle on aerial plant surfaces; suberin, which results from co-polymerization of lipid and phenolic derivatives and which regulates water movement in roots; and lignin, which is synthesized from phenolic precursors via the phenylpropanoid pathway and which reinforces secondary cell walls to enhance long-distance water transport and erect growth^3^,^4^,^5^. The metabolism of phenolic compounds was therefore likely a critical innovation for land colonization and, subsequently, the evolution and radiation of vascular plants through the development of lignified tissues^6^,^7^. Lignin-like polymers were recently identified in algae^8^,^9^, and phenylalanine/tyrosine ammonia lyases able to perform the first deamination step required for the synthesis of lignin precursors (that is, monolignols) from aromatic amino acids are present in algae and microorganisms^10^. However, orthologs of the next enzymes reported to frame the phenolic pathway in seed plants have not been found in algae, although they are present in bryophytes (mosses, liverworts and hornworts), the extant relatives of the earliest diverging land plants^6^,^11^,^12^, which played a major role in Earth's oxygenation^13^. Mosses do not have true vasculature and are considered to be non-lignified plants^14^, but current perspectives suggest the possibility of a pre-lignin pathway arising in early non-vascular plants during the first stages of the transition from water to land^3^.

To address this idea, we investigated the function of a moss cytochrome P450 enzyme, in the CYP98 family, the homologue of which in flowering plants (angiosperms) catalyses the first irreversible step committed to the biosynthesis of the major monolignols. CYP98 enzymes from angiosperms catalyse the *meta*-hydroxylation of the phenolic ring of the phenylpropane C5-C3 unit of *p*-coumaric acid^15^,^16^,^17^ (Fig. 1). The substrate for this *meta*-hydroxylation reaction, resulting in the formation of a caffeoyl derivative, is not free *p*-coumaric acid, but an ester of shikimic acid^17^,^18^. This implies a coupling, in angiosperms, of monolignols production to the plastidial shikimate pathway that forms aromatic amino acids^19^. *CYP98* inactivation in the model angiosperm *Arabidopsis thaliana* results in a severe suppression of growth and fertility^16^,^20^.

We show here that deletion of the single copy of *CYP98* present in the genome of the model moss *Physcomitrella patens* prevents the moss gametophore development and cuticle formation. Our phenolic profiling indicates that the moss pre-lignin pathway mainly leads to the formation of soluble caffeoyl-threonic acids, whereas free caffeic acid or other caffeic acid conjugates cannot be detected. Accordingly, PpCYP98 catalyses the formation of caffeoyl-threonic acids *in vitro*. We show that the cuticle impermeability is strongly decreased in the Δ*PpCYP98* mutants and can be restored together with plant growth by addition of caffeic acid. Our results thus demonstrate an essential role of the moss ancestral phenolic metabolism for plant erect growth and cuticle impermeability. These results are consistent with its proposed importance in plants terrestrialization.

## Results

### *PpCYP98* expression in developing gametophore and sporophyte

**Figure 1:**
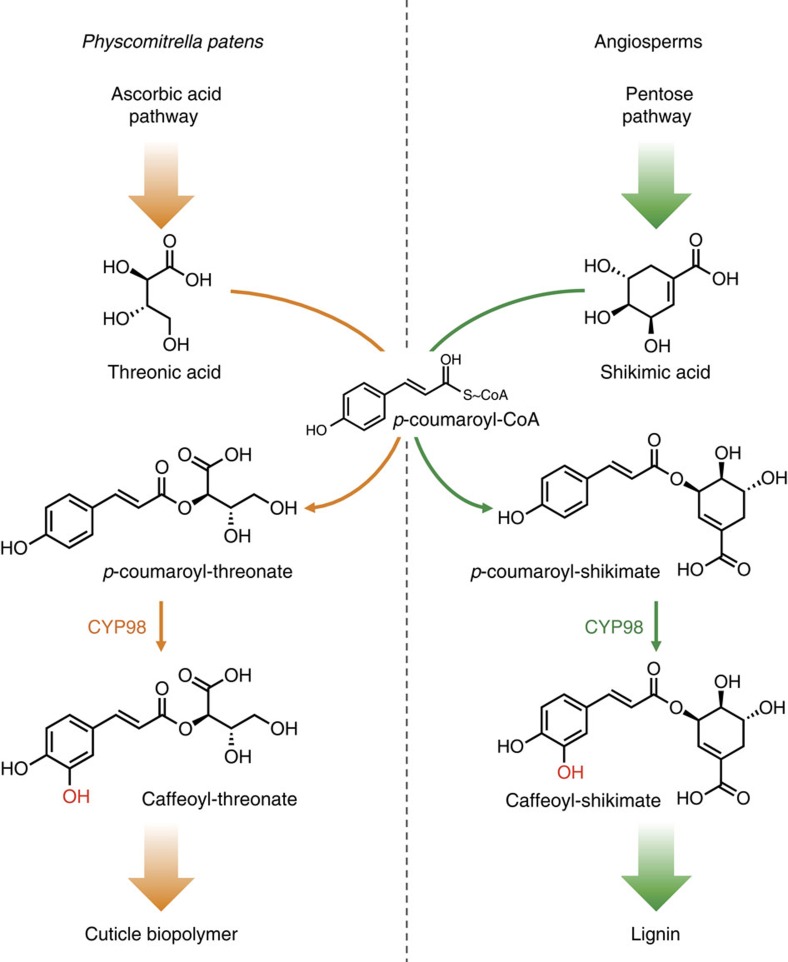
The hydroxycinnamoyl-shikimate pathway in angiosperms and the *P. patens* hydroxycinnamoyl-threonate pathway established in this study. The figure shows *p*-coumaroyl-2-theonate as PpCYP98 substrate, but *p*-coumaroyl-4-threonate is also converted by the enzyme. The *p*-coumaroyl-shikimate molecule is drawn according to the structure experimentally determined by Levsh *et al*.^39^.

**Figure 2:**
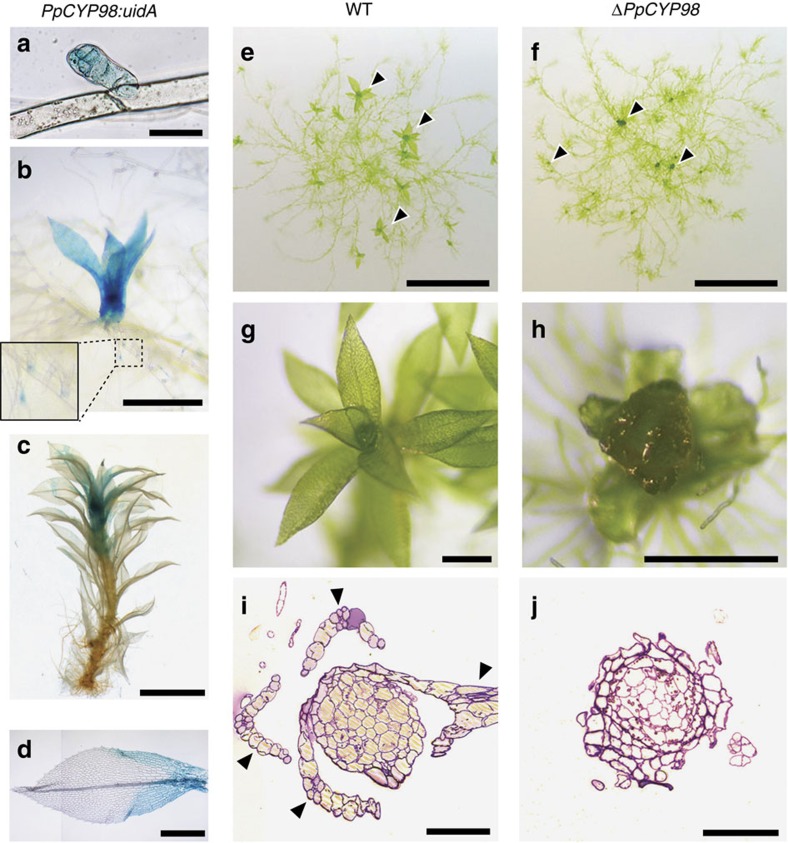
*PpCYP98* is essential for *P. patens* gametophore development. (**a**–**d**) GUS staining pattern in *PpCYP98:uidA* lines indicated a prominent expression in developing gametophores. (**a**) Bud (emerging gametophore). Scale bar, 50 μm. (**b**) Young gametophores. Scale bar, 0.5 mm. (**c**) One-month-old gametophore. Scale bar, 2 mm. (**d**) Apical leaf from 1-month-old gametophore (joined pictures). No GUS staining is visible in the midrib. Scale bar, 0.5 mm. (**e**–**j**) Δ*PpCYP98* mutants fail to develop normal gametophores. (**e**,**f**) Six-week-old colonies grown on agar plates. Arrowheads indicate gametophores. Scale bars, 5 mm. (**g**–**h**) Close-up views of gametophores. Scale bars, 0.5 mm. (**i**,**j**) Toluidine blue-stained cross section of gametophores. Arrowheads indicate phyllids in the WT. Scale bars, 0.1 mm.

A single-copy *CYP98* gene is present in the genome^6^ of the moss *P. patens* (*PpCYP98; CYP98A34; Pp3c22_19010V3.1*). Based on a transcriptome atlas^21^, the *PpCYP98* gene is mostly expressed in above-ground haploid gametophytic and diploid sporophytic tissues (Supplementary Fig. 1). An assessment of the pattern of expression of *PpCYP98* as determined after insertion of the *uidA* reporter gene encoding a β-glucuronidase (GUS) protein downstream of the *PpCYP98* gene in transgenic moss lines (Supplementary Fig. 2) confirmed the transcriptome data, and revealed prominent expression in erect aerial organs (Fig. 2a–d; Supplementary Fig. 2). The highest *PpCYP98* expression was observed in developing gametophores (Fig. 2a–c) and newly emerged and elongating phyllids (leaf-like structures; Fig. 2d), reproductive organs (gametangia: male antheridia and female archegonia), embryos and developing sporophytes including immature spores (Supplementary Fig. 2). Low levels of expression were also detected in the haploid filamentous protonema growing in direct contact with the wet substrate (Fig. 1b; Supplementary Fig. 1).

### *PpCYP98* prevents organ fusion

Three independent Δ*PpCYP98* deletion lines were generated (Supplementary Fig. 3) and showed no evidence of altered growth of the protonema filaments (Fig. 2e,f; Supplementary Fig. 4), but consistently exhibited aborted gametophore formation, associated with evidence of organ fusion, thereby precluded from forming gametangia and from sexual reproduction (Fig. 2g–j). Similar phenotypes have been observed in mutants of seed plants that are impaired in biosynthesis of the lipid biopolymer, cutin^22^,^23^.

### *PpCYP98* controls the formation of caffeoyl-threonic acids

**Figure 3:**
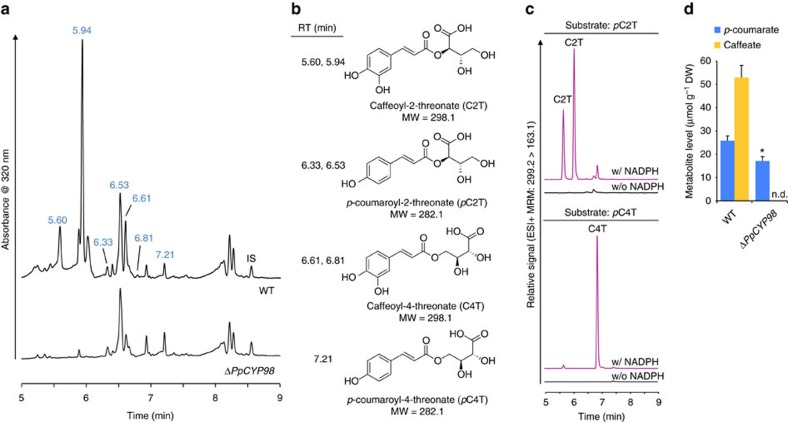
PpCYP98 is a phenolic ring *meta*-hydroxylase and uses esters of threonic acid as substrates. (**a**) Ultraviolet chromatogram showing the absence of major peaks in the Δ*PpCYP98* mutant gametophore crude extract. IS, internal standard (morin). (**b**) Names and structures of molecules at the indicated retention times (RT). (**c**) PpCYP98-dependent conversion of *p*-coumaroyl-2-threonate (*p*C2T) and *p*-coumaroyl-4-threonate (*p*C4T) esters into corresponding caffeoyl threonate esters (C2T and C4T). Control reactions without NADPH were concurrently analysed. Molecules were detected using dedicated multiple reaction monitoring (MRM) methods. Note that two caffeoyl-2-threonate isomers are produced from the two *p*-coumaroyl-2-threonate isomers present in the synthetic substrate, shown in Supplementary Fig. 6. (**d**) Acid hydrolysis of crude extracts demonstrates the total absence of caffeate in gametophores of the Δ*PpCYP98* mutants. Results are the mean+standard error from three independent biological samples for WT and three independent mutant lines. Asterisk indicates a significant difference between mutants and WT (*P*-value=0.037; two-tailed Student's *t*-test for samples of unequal variance).

Comparing profiles of soluble phenolic compounds extracted from the gametophores, we observed a complete absence in the mutant of several major compounds present in the wild type (WT; Fig. 3a). Based on mass and induced fragmentation patterns, the missing compounds were identified as isomers of caffeoyl-threonic acid (Fig. 3b; Supplementary Fig. 5, Supplementary Table 1). Other major phenolic compounds with slightly altered abundances in the mutant were subsequently assigned *p*-coumaroyl-threonate structures (Fig. 3b; Supplementary Fig. 6, Supplementary Table 1). This suggested that *p*-coumaroyl-threonic acid might undergo CYP98-dependent *meta*-hydroxylation in moss. To test this hypothesis, the enzyme was expressed in yeast (Supplementary Fig. 7) and was shown to convert chemically synthesized *p*-coumaroyl-2-threonic and *p*-coumaroyl-4-threonic acid substrates (Supplementary Note 2) into caffeoyl-2-threonic and caffeoyl-4-threonic acids, respectively (Fig. 3c).

Neither caffeic acid nor caffeoyl-shikimate/quinate was detected in moss crude extracts (Supplementary Table 1; Supplementary Fig. 8). To identify other potential caffeoyl derivatives in the extract, the latter was submitted to acid hydrolysis before liquid chromatography–mass spectrometry (LC–MS)/MS analysis, but no trace of caffeoyl residues was found in the mutant (Fig. 3d; Supplementary Table 2). *PpCYP98* deletion thus abolished the formation of free and bound caffeic acid.

### *PpCYP98* is required for the moss cuticle formation

**Figure 4:**
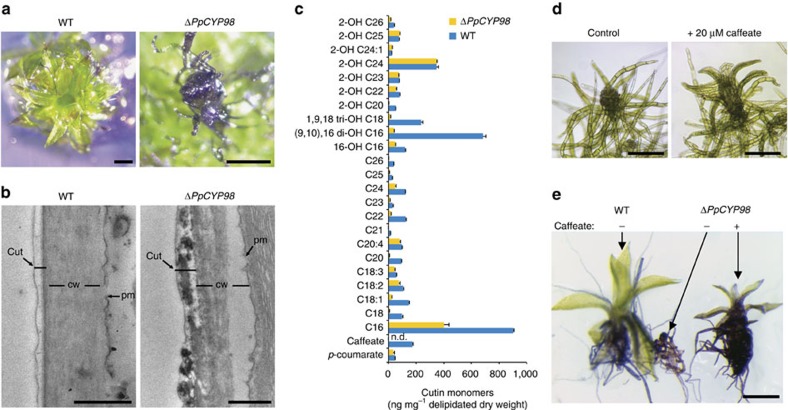
*PpCYP98* produces cutin caffeoyl units and is critical for cutin formation in *P. patens*. (**a**) Toluidine blue permeability staining indicates a cuticle defect in the Δ*PpCYP98* mutant gametophore. Scale bars, 0.5 mm. (**b**) Transmission electron micrographs of the phyllid outer cell surface showing alteration of the Δ*PpCYP98* mutant cuticle layer. cut, cuticle; cw, cell wall; pm, plasma membrane. Scale bars, 0.5 μm. (**c**) Comparative analysis of WT and mutant cutin gametophore composition. Results are the mean+standard error from three independent biological samples for WT and three independent lines for the mutant. n.d., not detected. (**d**) Exogenous caffeate supply (20 μM) restores growth of the Δ*PpCYP98* mutant gametophore. Scale bars, 0.2 mm. (**e**) Exogenous caffeate (20 μM) supply restores cuticle impermeability to toluidine blue of the mutant phyllids. Scale bar, 0.5 mm.

The organ fusion phenotype of the Δ*PpCYP98* mutant suggested impaired formation of the cuticle. This was further indicated by greatly increased surface permeability of the mutant gametophore to toluidine blue stain, compared with WT (Fig. 4a, Supplementary Fig. 9), and by transmission electron microscopy, which revealed perturbation of the integrity of the mutant cuticle (Fig. 4b). The composition of the biopolymer, cutin, the main constituent of cuticle, was therefore investigated. A recent study described the presence of a high proportion of phenolic residues in the cutin of *P. patens*^24^. Consistent with our observations, the cutin of the Δ*PpCYP98* mutant was devoid of caffeate residues (Fig. 4c) and, strikingly, also showed a substantial decrease in native and oxygenated C16 and C18 fatty acids monomers. This decrease in fatty acid content did not reflect fatty acid hydroxylase activity of the *PpCYP98* enzyme, which we determined did not catalyse C16 palmitic acid oxygenation (Supplementary Fig. 10). We therefore inferred that caffeic acid, or its derivatives, plays a key role in the cell-wall anchoring or structure of the cutin polymer in *P. patens*, or contributes to its regulation. Consistent with this hypothesis, addition of caffeic acid to the growth medium of the Δ*PpCYP98* mutant partially restored both plant growth and impermeability of the cuticle to toluidine blue (Fig. 4d,e; Supplementary Fig. 11).

## Discussion

Taken together, the free phenolic and polymer analyses indicate that caffeic acid derivatives are the major products of the phenolic metabolism in the moss *P. patens*. Our data also demonstrate that this moss ‘pre-lignin' pathway is crucial for the formation of a cuticular biopolymer and that caffeic acid production is a limiting factor in its biosynthesis. Unexpectedly, the substrate for the *meta*-hydroxylation reaction to form caffeic acid does not appear as free *p*-coumaric acid or *p*-coumaroyl-shikimate, but rather as *p*-coumaroyl-threonate. Threonic acid is a product of ascorbate catabolism^25^,^26^ and so we conclude that the formation of caffeic acid in moss is coupled to the hexose-derived ascorbate pathway, rather than the plastidial pentose phosphate-derived shikimate pathway, as is reported for vascular plants (Fig. 1). The importance of ascorbate has been revealed by the conservation of three different biosynthesis pathways in *P. patens*^27^. Stresses, including light and desiccation, are likely to regulate the formation of this biopolymer, and possibly the cuticle caffeic acid content, thereby increasing its antioxidant and ultraviolet-screening properties. The composition of the moss cuticle, namely its associating oxygenated phenolics and fatty acids, is more reminiscent of the polyester suberin than of cutin from seed plants^5^. It clearly confers surface protection and impermeability, and is also likely to contribute to the erect growth and rigidity of the moss gametophore. It may in particular contribute to the rigidity of the gametophore phyllid sheets, which are formed from a single-cell layer^24^. It has been hypothesized that cutin and suberin might share a common evolutionary origin^4^. Our data suggest that the lipid–phenolic matrix present in *P. patens* may constitute an extant representative of the common ancestor of the suberin, cutin and lignin polymers that have been described associated with highly differentiated vegetative tissues in more recently diverged plant lineages. This ancestral structure was presumably critical for the plant transition from water to land, and suggest new strategies for engineering biopolymers to enhance stress tolerance in vascular plants.

## Methods

### Plant material and growth conditions

*P. patens* (Hedw.) Bruch & Schimp. strain Grandsen^6^ was cultured in liquid or on solid Knop medium^28^ supplemented with 50 μmol l^−1^ H_3_BO_3_, 50 μmol l^−1^ MnSO_4_, 15 μmol l^−1^ ZnSO_4_, 2.5 μmol l^−1^ KI, 0.5 μmol l^−1^ Na_2_MoO_4_, 0.05 μmol l^−1^ CuSO_4_ and 0.05 μmol l^−1^ CoCl_2_. Medium was solidified with 12 g l^−1^ purified agar (OXOID, Thermo Scientific). Plants were kept at 23 °C under 16/8 h day/night cycle, light intensity set to 70 μmol m^−2^ s^−1^, and liquid cultures were upheld under constant agitation for proper aeration. Protonema material was grown in liquid cultures and maintained by weekly tissue disruption and subculturing. Gametophores were propagated on agar plates. In some cases, gametophores liquid cultures were established by soft tissue disruption (∼5 s) of individual gametophores. Sporophyte formation was induced in agar plate-grown gametophores by reducing day length and temperature as reported before^29^.

### Generation of transgenic lines

To generate the Δ*PpCYP98* knockout mutants, two 750 bp genomic regions were PCR-amplified from *P. patens* genomic DNA and assembled with the *nptII* selection cassette into a PCR-linearized pGEM-T vector via GIBSON cloning^30^ (Supplementary Fig. 3, Supplementary Table 3 for primers). The *PpCYP98* disruption construct was excised from the vector backbone by *Bam*HI digestion, using restriction sites introduced during PCR. Twenty-five microgram of linearized construct were used for PEG-mediated transfection of *P. patens* protoplast^31^. Transformants were selected on Knop plates supplemented with 25 mg l^−1^ geneticin (G418).

For *PpCYP98:uidA* reporter lines, two 800 bp genomic regions framing the *PpCYP98* STOP codon were PCR-amplified from *P. patens* genomic DNA and assembled with the *uidA* reporter gene into a PCR-linearized pGEM-T vector via GIBSON cloning^30^ (Supplementary Fig. 2, Supplementary Table 3). A linker DNA sequence was introduced during PCR to limit GUS protein hindrance on PpCYP98 activity (Supplementary Fig. 2). The *PpCYP98:uidA* construct was excised from the vector backbone by *Eco*RI digestion, using restriction sites introduced during PCR. Twenty-five micrograms of linearized construct were used for transfection of *P. patens* protoplast^31^. The *PpCYP98:uidA* construct does not harbour a selection cassette and was therefore co-transfected with the pRT101 plasmid^32^ containing the *nptII* selection cassette. Transformants were selected on Knop plates supplemented with 25 mg l^−1^ geneticin (G418).

### Molecular characterization of transgenic lines

Following the selection process, a previously established direct-PCR protocol^33^ was implemented to identify transformants with proper genomic integration of the DNA construct. Briefly, one gametophore or 2–3 protonema filaments were incubated for 15 min at 45 °C in a DNA extraction buffer (9.1 g l^−1^ Tris-HCl pH 8.8, 2.6 g l^−1^ (NH_4_)_2_SO_4_, 0.1 ml l^−1^ Tween 20). PCR reactions were performed with the Phire II polymerase (Thermo Scientific) and 2.5 μl of the DNA extract in 15 μl reaction. Both 5′ and 3′ integrations were verified using appropriate PCR strategy and primers (Supplementary Figs 2 and 3, Supplementary Table 3).

Δ*PpCYP98* lines identified by direct-PCR were further validated at the molecular level by conventional PCR with reverse transcription (Supplementary Fig. 3C). To this end, total RNA was isolated from ∼8 mg of lyophilized 3-day-old protonema material using TriReagent (Sigma-Aldrich). Twenty micrograms of RNA were treated with 5U of RQ1 DNaseI (Promega) and subsequently purified with the Nucleospin RNA clean-up XS kit (Macherey-Nagel). One microgram of DNaseI-treated RNA was reverse-transcribed with oligo(dT)_23_V and the Superscript III enzyme (Thermo Scientific) in 20 μl reaction. *PpCYP98* transcripts were amplified from 1 μl of cDNA using the Phire II polymerase (Thermo Scientific) in 20 μl reaction. The constitutively expressed *L21* gene (Pp1s107_181V6.1), encoding a 60S ribosomal protein, was used as reference gene.

The number of Δ*PpCYP98* lines was narrowed down after transcript analysis; remaining lines were subjected to transgene copy analysis by quantitative PCR as described before^34^. Genomic DNA was isolated using a protocol adapted from Edwards *et al*.^35^. Briefly, nucleic acids were extracted from 5 mg of 3-day-old lyophilized protonema material with 500 μl of lysis buffer (200 mM Tris-HCl pH 8.0, 250 mM NaCl, 25 mM EDTA, 0.5% SDS) and thorough agitation. Nucleic acids were purified by addition of a 500 μl of a phenol:chloroform (1:1) solution (pH 8.0) followed by a precipitation with sodium acetate and isopropanol. DNA pellets were washed with 75% ethanol before solubilization in a 5 mM Tris pH 8.5 solution. Samples were further treated with a RNase A/T1 mix (Thermo Scientific) to remove RNA. DNA was re-purified with a phenol:chloroform step as described above. Typical yields were ∼0.5 μg DNA per mg of dry plant material. Quantitative PCR reactions were run on a LightCycler 480 II device (Roche) in 10 μl comprising 1 ng genomic DNA reaction, 250 nM of each primer and 1X LightCycler 480 SYBR Green I Master mix (Roche). Reactions were performed in triplicate and crossing points were determined using the manufacturer software. Both the 5′- and 3′-homologous regions were targeted using specific primers (Supplementary Fig. 3, Supplementary Table 3). The single-copy gene *PpCLF* (Pp1s100_146V6.1) was amplified using two primer pairs and served as an internal standard for input amount normalization. Transgene copy number was determined by comparing relative values of the tested genomic segment in transgenic lines with those of the WT. Three Δ*PpCYP98* lines with a single transgene copy (Supplementary Fig. 3) were kept for subsequent phenotypic and metabolic analyses.

### GUS staining

Plant tissues were vacuum infiltrated with X-Gluc solution (1 mM 5-bromo-4-chloro-3-indolyl-β-glucoronide, 100 mM sodium phosphate and pH 7.0) and incubated at 37 °C for 24 h. The samples were fixed in 5% v/v formaldehyde, 5% v/v acetic acid, 100 mM sodium phosphate and pH 7.0 for 30 min. Chlorophyll was extracted with an ethanol series (30, 50, 70 and 100%).

### Recombinant proteins production in yeast and enzyme assays

Procedures were adapted from Liu *et al*.^36^. Coding sequence of the *P. patens PpCYP98* gene was optimized for yeast expression (sequences available in Supplementary Note 1) and synthesized by GeneCust Europe (Dudelange, Luxembourg) with addition of *Bam*HI and *Kpn*I restriction sites at the 5′ and 3′ termini, respectively. *Bam*HI/*Kpn*I sites were used for subcloning CDS into the yeast expression plasmid pYeDP60.

The *Saccharomyces cerevisiae* strain WAT11, expressing the *ATR1* cytochrome P450 reductase from *Arabidopsis thaliana* under the control of the galactose-inducible promoter *GAL10-CYC1* (ref. 37), was transformed with the pYeDP60:PpCYP98 plasmid and selected on minimum SGI medium (1 g l^−1^ bactocasamino acids, 7 g l^−1^ yeast nitrogen base, 20 g l^−1^ glucose and 40 mg l^−1^ L-tryptophan). Isolated colonies were chosen for initiating SGI liquid cultures and were grown for 18 h at 28 °C under 160 r.p.m. agitation. Ten millilitres of SGI culture were used to inoculate 200 ml of YPGE medium (10 g l^−1^ bactopeptone, 10 g l^−1^ yeast extract, 5 g l^−1^ glucose and 3% ethanol by volume). Following a 30 h growth period at 28 °C under 160 r.p.m. agitation, production of recombinant proteins was induced by supplementing the growth medium with 10 ml of 200 g l^−1^ sterile galactose solution. Proteins production phase was performed at 20 °C for 16 h.

Yeast cells were harvested by centrifugation at 7,500*g* for 10 min at 4 °C, washed with TEK buffer (50 mM Tris-HCl pH 7.5, 1 mM EDTA, 100 mM KCl) and resuspended in 2 ml of TES buffer (50 mM Tris-HCl pH 7.5, 1 mM EDTA, 600 mM sorbitol) supplemented with 5 mM 2-mercaptoethanol and 10 g l^−1^ bovine serum albumin, fraction V (buffer A). Cell suspensions were transferred to 50 ml conical tubes and homogenized with 0.5 mm glass beads by handshaking five times for 1 min. Beads were washed twice with 30 ml of buffer A. Cell debris and remaining glass beads were removed from the pooled lysates by a 20 min centrifugation step at 7,500*g* and 4 °C. Supernatants were filtrated on Miracloth (22–25 μm pore size, Calbiochem, MD, USA) and microsomal fractions were purified by differential centrifugation (100,000*g*, 4 °C, 1 h). Pelleted microsomes were resuspended in TEG buffer (50 mM Tris-HCl pH 7.5, 0.5 mM EDTA, 30% glycerol by volume) with a Potter-Elvehjem homogenizer. Microsomes preparations containing PpCYP98 recombinant proteins were stored at −20 °C until processing.

Enzyme assays were performed in 50 mM potassium phosphate buffer (pH 7.4) containing 100 μM substrate, 10 pmol of PpCYP98 recombinant enzyme and 500 μM NADPH in a final volume of 200 μl. Reactions were initiated by addition of the NADPH, incubated at 28 °C in the dark for 30 min and terminated with 1/10 50% acetic acid and 4/10 acetonitrile. Microsomal membranes were spun down by centrifugation (13,000*g*, 10 min, 4 °C). Reaction products were analysed from supernatants by Ultra Performance Liquid Chromatography (UPLC)-MS/MS as described below.

### Metabolic profiling of *Physcomitrella patens*

Protonema material was harvested 3 days after tissue disruption. Gametophores grown in liquid medium were harvested 1 month after the last disruption and 1 week after medium change. *P. patens* tissues were collected by pouring the culture onto a sieve (100 μm pore size) and were thoroughly rinsed with distilled water, quickly blotted on paper towel and finally snap-frozen in liquid nitrogen. Tissues were lyophilized overnight before grinding with 5 mm steel balls and a Tissuelyser II (Qiagen) operated at 30 Hz for 2 min.

Metabolites were extracted with a previously described methanol:chloroform:water protocol^38^. Briefly, 400 μl of methanol containing 10 μM morin as an internal standard were added to 10 mg of lyophilized ground plant material. After agitation at 1,500 r.p.m. for 1 h, 200 μl of chloroform were added and samples were shaken for five additional minutes. Phase separation was induced by adding 400 μl of ultra-pure water followed by vigorous agitation and centrifugation (15,000*g*, 4 °C, 15 min). Supernatants were transferred to clean microtubes and constituted the crude extracts. To hydrolyse ester bonds of hydroxycinnamic acid conjugates, 250 μl of crude extract were dried under reduced pressure. Dry residues were resuspended in a 50% methanol solution containing 2 N HCl and 2.5 mg ml^−1^ ascorbic acid as an antioxidant. Acid hydrolysis was performed at 80 °C for 2 h.

Metabolites separation and detection were carried out on an Acquity UPLC (Waters corp.) coupled to a photodiode array detector (Waters corp.) and a Quattro Premier XE tandem-mass spectrometer (Waters corp.) equipped with an electrospray ionization source. Ten microliters of extract were injected onto a UPLC BEH C18 column (100 × 2.1 mm, 1.7 μm; Waters) outfitted with a pre-column and operated at 35 °C. Metabolites chromatography was performed at a 0.35 ml min^−1^ flow rate with a mixture of 0.1% formic acid in water (solvent A) and 0.1% formic acid in acetonitrile (solvent B) according to the following programme: initial, 98% A; 11.25 min, 0% A, curve 8; 12.75 min, 0% A, curve 6; 13.50 min, 98% A, curve 6; 15 min, 98% A. Nitrogen was used as the drying and nebulizing in-source gas. The nebulizer gas flow was set to 50 l h^−1^, and the desolvation gas flow was set to 900 l h^−1^. The interface temperature was set to 400 °C, and the source temperature to 135 °C. Capillary voltage was set to 3.4 kV. Data acquisition and analysis were performed with the MassLynx v4.1 software (Waters corp.). Metabolites were ionized in positive mode and detected using dedicated multiple reaction monitoring methods (Supplementary Table 4). The QuanLynx module of MassLynx was executed to integrate peaks and to report corresponding areas. Peak areas were normalized to plant dry weight and internal standard level (morin), leading to relative levels. External calibration curves of authentic standards were employed for the absolute quantification of hydroxycinnamic acid after acid hydrolysis.

### *In vitro* assay for palmitic acid conversion

The radiolabelled substrate was dissolved in ethanol that was evaporated before the addition of microsomal fraction into the glass tube. Resolubilization of the substrates was confirmed by measuring the radioactivity of the incubation media. The standard assay (0.1 ml) contained 20 mM sodium phosphate (pH 7.4), 1 mM NADPH, radiolabelled substrate (100 μM) and 35 μl of microsomal fraction. The reaction was initiated by the addition of NADPH and was done in a bathwater 20 min at 27 °C under agitation. Incubation media were directly spotted on thin layer chromatography (TLC) plates. For separation of metabolites from residual substrate, TLC plates were developed with a mixture of diethyl ether/light petroleum (boiling point, 40–60 °C)/formic acid (50:50:1, v/v/v). The plates were scanned with a radioactivity detector (Raytest Rita Star).

### Transmission electron microscopy

Samples were fixed overnight in 2% glutaraldehyde and were then treated for 2 h with 2% uranyl acetate 2% (w/v) and then stained with osmium tetroxide 0.1% (v/v) in 150 mM phosphate buffer, pH 7.2. Samples were dehydrated through an ethanol series and infiltrated with EPON812 medium grade resin (Polysciences). Polymerization was allowed for 48 h at 60 °C. Ultrathin sections (70 microns) were cut using an ultracut E microtome (Reichert) and collected on grids coated with formvar (electron microscopy sciences (EMS)). Samples were visualized with a Hitachi H-7500 electron microscope operating at 80 kV.

### Permeability test

Tissue permeability was assessed by immersing plants into a 0.05% toluidine blue solution for 2 min. Samples were subsequently thoroughly washed with distilled water until the washing solution was clear.

### Cutin monomers analysis

Cutin monomers analysis was performed on the same plant material as used for metabolic analysis (that is, lyophilized gametophores grown in liquid medium). For each sample, ∼200 mg of ground, lyophilized moss tissue was added to a pre-weighed 40 ml glass vial for processing. The tissue was delipidated by extensive washing with a series of solvents, each containing 0.01% butylated hydroxytoluene, as follows.

A total of 40 ml of isopropanol pre-heated to 85 °C was added to each sample and incubated at 85 °C for 15 min, then after cooling to room temperature, the samples were shaken at 250 r.p.m. for 1 h and centrifuged at 1,500*g* for 5 min to pellet most of the tissue. The supernatant was removed with a glass Pasteur pipette and filtered through a paper filter (Whatman #1) to collect the suspended tissue particles, which were then scraped off the paper and added back to the sample vial. Three more washes (agitation, centrifugation and filtering) were performed, adding 30 ml of room temperature isopropanol each time. The tissue was then washed with the following series of solvents: 2:1 chloroform: methanol (repeated once), 1:2 chloroform: methanol, 1:1 chloroform: methanol, 1:2 chloroform: methanol, pure methanol. The samples were agitated with each of these solvents at 250 r.p.m. for at least 2 h and up to overnight, with centrifugation and filtration (as described above) after each wash. The tissue was then dried under a stream of nitrogen for 20 min and freeze dried overnight. The vial was weighed to determine the dry, delipidated tissue weight.

A total of 8 ml of reaction media (12:3:5 methanol: methyl acetate: 25% sodium methoxide) and 100 μg of each of pentadecalactone and heptadecanoate (internal standards) were added to each sample, and the mixture was then heated at 60 °C overnight to depolymerize the cutin. The samples were then cooled to room temperature and 16 ml dichloromethane, 2 ml glacial acetic acid and 4 ml 0.9% NaCl in 100 mM Tris (pH 8.0) was added to each and vortexed to mix. Two phases were separated by centrifugation for 2 min at 1,500*g*. The lower phase was transferred to a new vial and 14 ml of 0.9% NaCl in 100 mM Tris (pH 8.0) was added and mixed by vortexing. Two phases were again separated by centrifugation (2 min at 1,500*g*) and the lower phase was transferred to a new vial, while the upper phase was discarded. Roughly 0.5 g of anhydrous sodium sulfate added to remove any residual water, and the solution then filtered through a paper filter (Whatman #1) into a clean vial.

An aliquot of each cutin sample was dried by heating at 40 °C under a stream of nitrogen, then derivatized with 50 μl each of pyridine and BSTFA (N,O-bis(trimethylsilyl)trifluoroacetamide) for 10 min at 90 °C, dried again by heating under nitrogen, and resuspended in 100 μl chloroform. The samples were analysed by gas chromatography (GC) using an Agilent GC 6850 with a Flame Ionization Detector. Compounds were identified based on a comparison of retention times with standards and also by performing GC–mass spectrometry (MS) using an Agilent GC 6890 coupled to a JEOL GC MATE II mass spectrometer. Cutin levels were normalized to the internal standards and the dry, delipidated tissue weights.

### Chemical complementation

For the chemical complementation of the Δ*PpCYP98* plants, caffeic acid (Sigma-Aldrich) was solubilized in absolute ethanol to prepare 100 mM stock solutions. Freshly prepared stock solutions were added to liquid cultures 14 days after the last disruption to a final concentration of 20 μM. After 14 days the medium was exchanged for fresh medium containing caffeic acid. Gametophore development was assessed after 14 and 28 days cultivation in presence of caffeic acid. Control cultures containing 0.05% ethanol were run in parallel.

### Chemical synthesis of *p*-coumaroyl-threonate esters

Detailed procedures are available in Supplementary Note 2.

### Data availability

The data that support the findings of this study are available from the corresponding authors upon request. The transgenic moss lines described in this study are deposited in the International Moss Stock Center (Ahttp://www.moss-stock-center.org/) with the accession numbers IMSC 40805-40814.

## Additional information

**How to cite this article**: Renault, H. *et al*. A phenol-enriched cuticle is ancestral to lignin evolution in land plants. *Nat. Commun.* **8**, 14713 doi: 10.1038/ncomms14713 (2017).

**Publisher's note**: Springer Nature remains neutral with regard to jurisdictional claims in published maps and institutional affiliations.

## Supplementary Material

Supplementary Information Supplementary Figures, Supplementary Tables, Supplementary Notes and Supplementary References

## References

[b1] Kenrick P. & Crane P. R. The origin and early evolution of plants on land. Nature 389, 33–39 (1997).

[b2] Bowman J. L. Walkabout on the long branches of plant evolution. Curr. Opin. Plant Biol. 16, 70–77 (2013). 23140608 10.1016/j.pbi.2012.10.001

[b3] Weng J. K. & Chapple C. The origin and evolution of lignin biosynthesis. New Phytol. 187, 273–285 (2010). 20642725 10.1111/j.1469-8137.2010.03327.x

[b4] Fich E. A., Segerson N. A. & Rose J. K. The plant polyester cutin: biosynthesis, structure, and biological roles. Annu. Rev. Plant Biol. 67, 207–233 (2016). 26865339 10.1146/annurev-arplant-043015-111929

[b5] Beisson F., Li-Beisson Y. & Pollard M. Solving the puzzles of cutin and suberin polymer biosynthesis. Curr. Opin. Plant Biol. 15, 329–337 (2012). 22465132 10.1016/j.pbi.2012.03.003

[b6] Rensing S. A. . The Physcomitrella genome reveals evolutionary insights into the conquest of land by plants. Science 319, 64–69 (2008). 18079367 10.1126/science.1150646

[b7] Tohge T., Watanabe M., Hoefgen R. & Fernie A. R. The evolution of phenylpropanoid metabolism in the green lineage. Crit. Rev. Biochem. Mol. Biol. 48, 123–152 (2013). 23350798 10.3109/10409238.2012.758083

[b8] Martone P. T. . Discovery of lignin in seaweed reveals convergent evolution of cell-wall architecture. Curr. Biol. 19, 169–175 (2009). 19167225 10.1016/j.cub.2008.12.031

[b9] Sorensen I. . The charophycean green algae provide insights into the early origins of plant cell walls. Plant J. 68, 201–211 (2011). 21707800 10.1111/j.1365-313X.2011.04686.x

[b10] Barros J. . Role of bifunctional ammonia-lyase in grass cell wall biosynthesis. Nat. Plants 2, 16050 (2016). 27255834 10.1038/nplants.2016.50

[b11] Nelson D. & Werck-Reichhart D. A P450-centric view of plant evolution. Plant J. 66, 194–211 (2011). 21443632 10.1111/j.1365-313X.2011.04529.x

[b12] Xu Z. . Comparative genome analysis of lignin biosynthesis gene families across the plant kingdom. BMC Bioinformatics 10, (Suppl 11): S3 (2009). 10.1186/1471-2105-10-S11-S3 PMC3226193 19811687

[b13] Lenton T. M. . Earliest land plants created modern levels of atmospheric oxygen. Proc. Natl Acad. Sci. USA 113, 9704–9709 (2016). 27528678 10.1073/pnas.1604787113 PMC5024600

[b14] Ligrone R., Ducket J. G. & Renzaglia K. S. Conducting tissues and phyletic relationships of bryophytes. Philos. Trans. R. Soc. Lond. B Biol. Sci. 355, 795–813 (2000). 10905610 10.1098/rstb.2000.0616 PMC1692789

[b15] Franke R. . Changes in secondary metabolism and deposition of an unusual lignin in the ref8 mutant of Arabidopsis. Plant J. 30, 47–59 (2002). 11967092 10.1046/j.1365-313x.2002.01267.x

[b16] Franke R. . The Arabidopsis REF8 gene encodes the 3-hydroxylase of phenylpropanoid metabolism. Plant J. 30, 33–45 (2002). 11967091 10.1046/j.1365-313x.2002.01266.x

[b17] Schoch G. . CYP98A3 from *Arabidopsis thaliana* is a 3′-hydroxylase of phenolic esters, a missing link in the phenylpropanoid pathway. J. Biol. Chem. 276, 36566–36574 (2001). 11429408 10.1074/jbc.M104047200

[b18] Coleman H. D., Park J. Y., Nair R., Chapple C. & Mansfield S. D. RNAi-mediated suppression of *p*-coumaroyl-CoA 3′-hydroxylase in hybrid poplar impacts lignin deposition and soluble secondary metabolism. Proc. Natl Acad. Sci. USA 105, 4501–4506 (2008). 18316744 10.1073/pnas.0706537105 PMC2393750

[b19] Schoch G. A. . The meta-hydroxylation step in the phenylpropanoid pathway: a new level of complexity in the pathway and its regulation. Environ. Chem. Lett. 4, 127–136 (2006).

[b20] Abdulrazzak N. . A coumaroyl-ester-3-hydroxylase insertion mutant reveals the existence of nonredundant meta-hydroxylation pathways and essential roles for phenolic precursors in cell expansion and plant growth. Plant Physiol. 140, 30–48 (2006). 16377748 10.1104/pp.105.069690 PMC1326029

[b21] Ortiz-Ramirez C. . A transcriptome atlas of *Physcomitrella patens* provides insights into the evolution and development of land plants. Mol. Plant 9, 205–220 (2016). 26687813 10.1016/j.molp.2015.12.002

[b22] Sieber P. . Transgenic Arabidopsis plants expressing a fungal cutinase show alterations in the structure and properties of the cuticle and postgenital organ fusions. Plant Cell 12, 721–738 (2000). 10810146 10.1105/tpc.12.5.721 PMC139923

[b23] Javelle M., Vernoud V., Rogowsky P. M. & Ingram G. C. Epidermis: the formation and functions of a fundamental plant tissue. New Phytol. 189, 17–39 (2011). 21054411 10.1111/j.1469-8137.2010.03514.x

[b24] Buda G. J. . An ATP binding cassette transporter is required for cuticular wax deposition and desiccation tolerance in the moss *Physcomitrella patens*. Plant Cell 25, 4000–4013 (2013). 24163310 10.1105/tpc.113.117648 PMC3877811

[b25] Green M. A. & Fry S. C. Vitamin C degradation in plant cells via enzymatic hydrolysis of 4-O-oxalyl-L-threonate. Nature 433, 83–87 (2005). 15608627 10.1038/nature03172

[b26] Debolt S., Melino V. & Ford C. M. Ascorbate as a biosynthetic precursor in plants. Ann. Bot. 99, 3–8 (2007). 17098753 10.1093/aob/mcl236 PMC2802977

[b27] Mueller S. J. . Quantitative analysis of the mitochondrial and plastid proteomes of the moss *Physcomitrella patens* reveals protein macrocompartmentation and microcompartmentation. Plant Physiol. 164, 2081–2095 (2014). 24515833 10.1104/pp.114.235754 PMC3982764

[b28] Reski R. & Abel W. O. Induction of budding on chloronemata and caulonemata of the moss, *Physcomitrella patens*, using isopentenyladenine. Planta 165, 354–358 (1985). 24241140 10.1007/BF00392232

[b29] Hohe A., Rensing S. A., Mildner M., Lang D. & Reski R. Day length and temperature strongly influence sexual reproduction and expression of a novel MADS-box gene in the moss *Physcomitrella patens*. Plant Biol. 4, 595–602 (2002).

[b30] Gibson D. G. . Enzymatic assembly of DNA molecules up to several hundred kilobases. Nat. Methods 6, 343–345 (2009). 19363495 10.1038/nmeth.1318

[b31] Hohe A. . An improved and highly standardised transformation procedure allows efficient production of single and multiple targeted gene-knockouts in a moss, *Physcomitrella patens*. Curr. Genet. 44, 339–347 (2004). 14586556 10.1007/s00294-003-0458-4

[b32] Girke T., Schmidt H., Zahringer U., Reski R. & Heinz E. Identification of a novel delta 6-acyl-group desaturase by targeted gene disruption in *Physcomitrella patens*. Plant J. 15, 39–48 (1998). 9744093 10.1046/j.1365-313x.1998.00178.x

[b33] Schween G., Fleig S. & Reski R. High-throughput-PCR screen of 15,000 transgenic Physcomitrella plants. Plant Mol. Biol. Rep. 20, 43–47 (2002).

[b34] Horst N. A. . A single homeobox gene triggers phase transition, embryogenesis and asexual reproduction. Nat. Plants 2, 15209 (2016). 27250874 10.1038/nplants.2015.209

[b35] Edwards K., Johnstone C. & Thompson C. A simple and rapid method for the preparation of plant genomic DNA for PCR analysis. Nucleic Acids Res. 19, 1349 (1991). 2030957 10.1093/nar/19.6.1349 PMC333874

[b36] Liu Z. . Evolutionary interplay between sister cytochrome P450 genes shapes plasticity in plant metabolism. Nat. Commun. 7, 13026 (2016). 27713409 10.1038/ncomms13026 PMC5059761

[b37] Urban P., Mignotte C., Kazmaier M., Delorme F. & Pompon D. Cloning, yeast expression, and characterization of the coupling of two distantly related *Arabidopsis thaliana* NADPH-cytochrome P450 reductases with P450 CYP73A5. J. Biol. Chem. 272, 19176–19186 (1997). 9235908 10.1074/jbc.272.31.19176

[b38] Renault H. . The Arabidopsis pop2-1 mutant reveals the involvement of GABA transaminase in salt stress tolerance. BMC Plant Biol. 10, 20 (2010). 20122158 10.1186/1471-2229-10-20 PMC2825238

[b39] Levsh O. . Dynamic conformational states dictate selectivity toward the native substrate in a substrate-permissive acyltransferase. Biochemistry 55, 6314–6326 (2016). 27805809 10.1021/acs.biochem.6b00887 PMC6276119

