# Supplementary material for: A phenol-enriched cuticle is ancestral to lignin evolution in land plants
Source: Nat Commun. 2017 Mar 8;8:14713. doi: 10.1038/ncomms14713 (PMC5344971; doi:10.1038/ncomms14713)
Supplement: Supplementary Information — Supplementary Figures, Supplementary Tables, Supplementary Notes and Supplementary References [file ncomms14713-s1.pdf]

Supplementary Figures

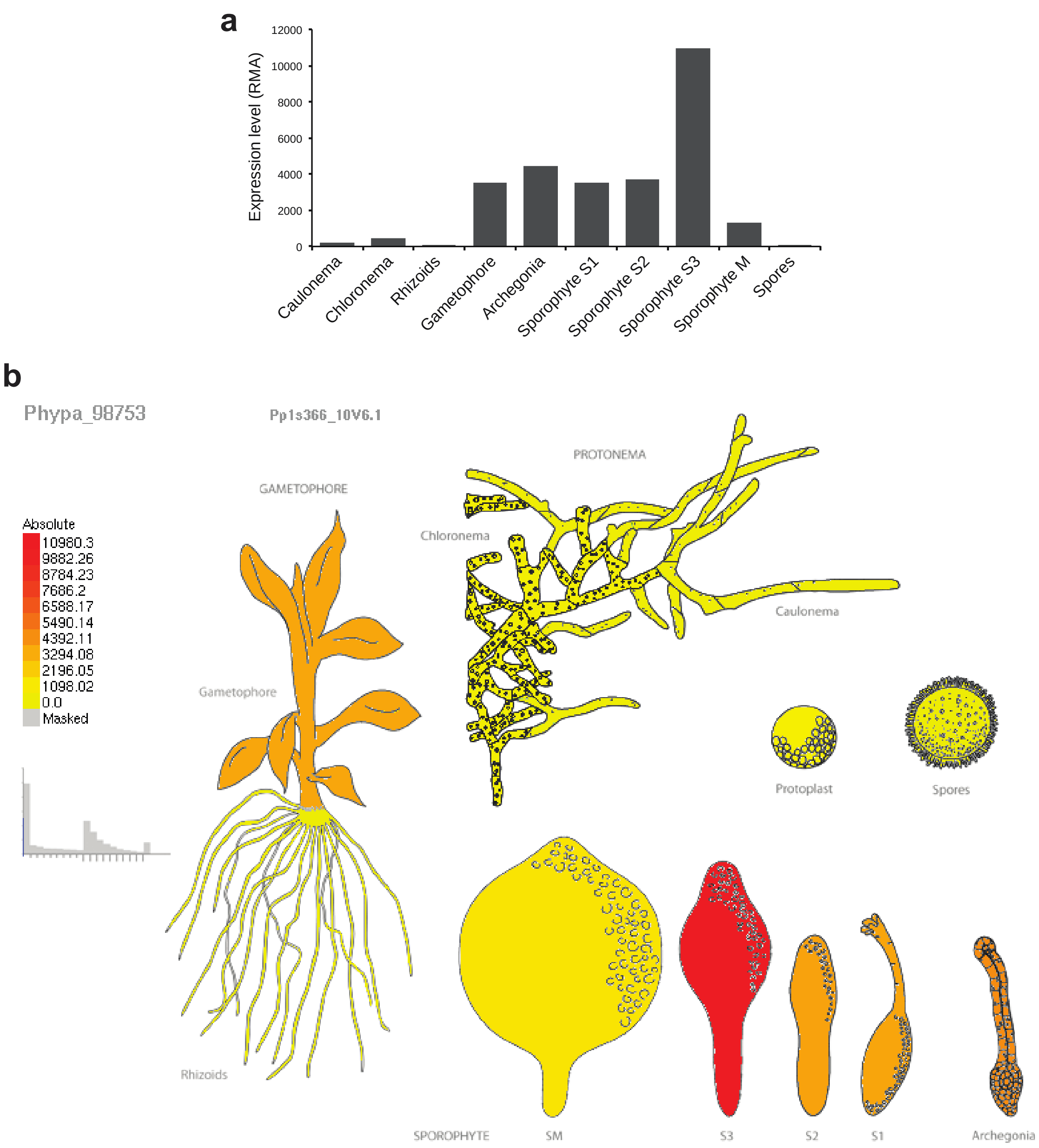

Supplementary Figure 1 | *PpCYP98* expression throughout the *Physcomitrella patens* life cycle. Data are derived from the *P. patens* transcriptome atlas[1]. (**a**) Bar chart representation. (**b**) Data visualization via the "Electronic Fluorescent Pictograph" (eFP) software[2].

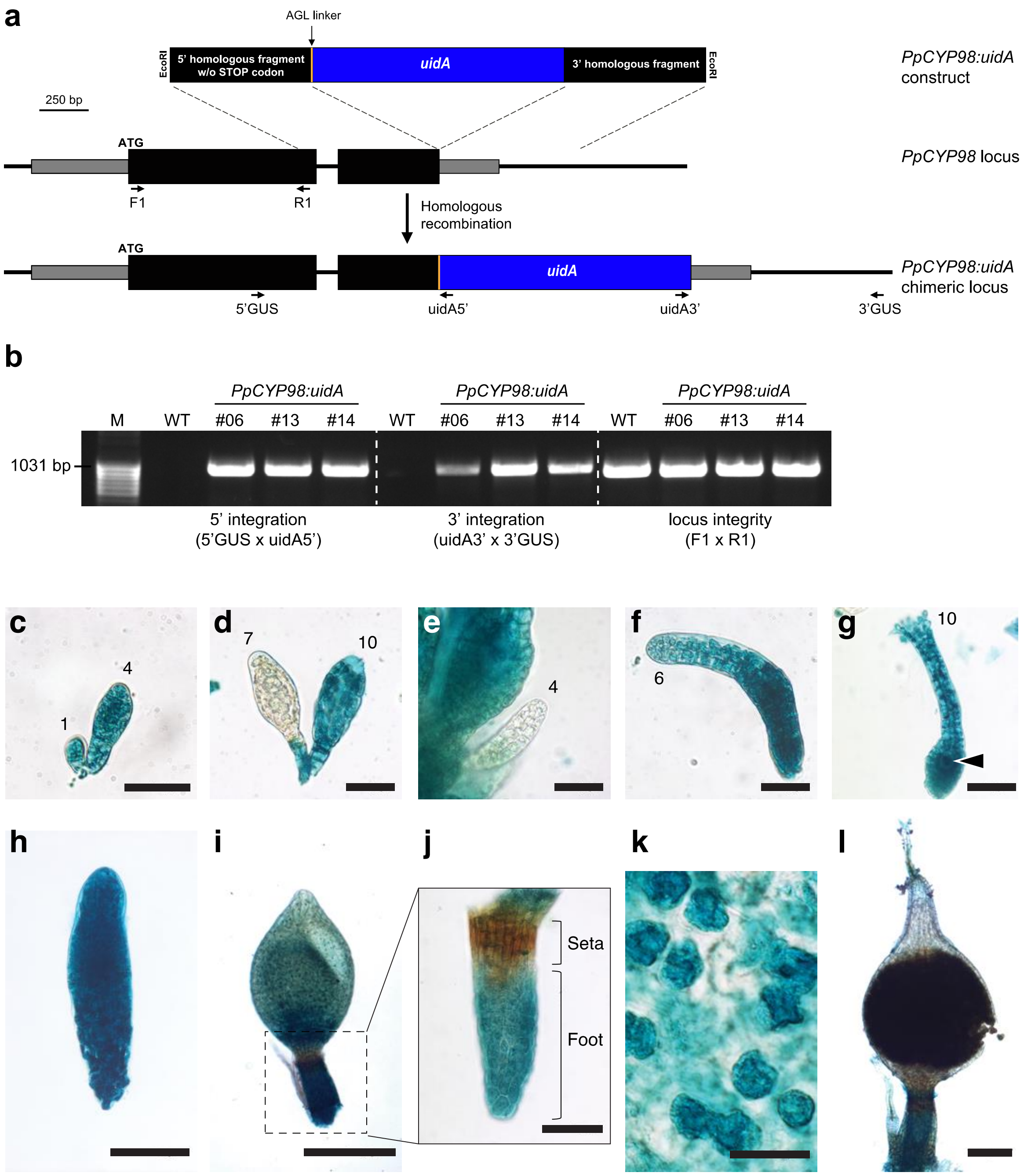

Supplementary Figure 2 | Expression of *PpCYP98* in *P. patens* reproductive tissues. (**a**) Homologous recombination-mediated strategy for *PpCYP98* fusion with the *uidA* reporter gene: the *uidA* gene preceded by a sequence encoding an alanine-glycine-leucine (AGL) linker was inserted in place of the *PpCYP98* STOP codon. (**b**) PCR validation of the correct integration of the construct in the *PpCYP98* genomic locus of the G418-selected transgenic lines. Primer hybridization sites are indicated in (**a**). WT, wild type; M, DNA size marker (MassRuler DNA Ladder Mix, Thermo Fisher Scientific). GUS staining of the *PpCYP98:uidA* lines was used to investigate *PpCYP98* expression in the gametangia and the sporophyte. (**c-d**) Antheridia. Scale bars, 40 µm. (**e-g**) Archegonia. Arrowhead in (**g**) points to the egg cell. Numbers indicate the developmental stage (1-10) of the gametangia according to[3]. Scale bars: C-D, 40 µm; E, 80 µm. (**h**) Embryo. Scale bar, 100 µm. (**i**) Mid-stage sporophyte. Scale bar, 400 µm. (**j**) Magnification of box in (**i**). The signal derived from the PpCYP98:GUS fusion protein is present in the foot but not in the differentiated seta. Scale bar, 100 µm. (**k**) Tetrads of immature spores. Scale bar, 20 µm. (**l**) Mature sporophyte. GUS signal is still detected in the metabolically active foot. Scale bar, 200 µm.

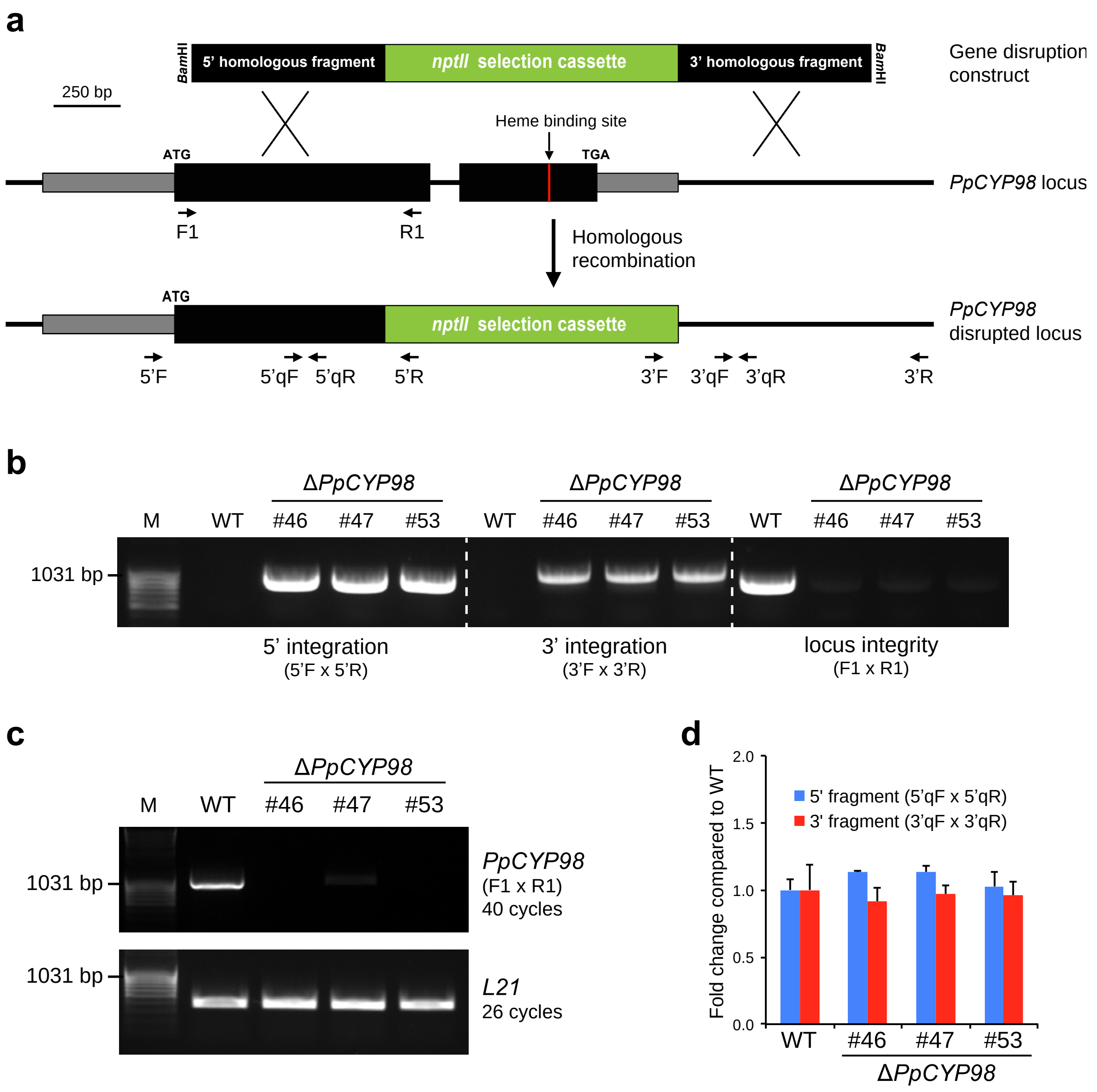

Supplementary Figure 3 | Strategy for *PpCYP98* inactivation and molecular characterization of the Δ*PpCYP98* mutants. (**a**) Homologous recombination-mediated strategy for *PpCYP98* gene disruption: a *PpCYP98* genomic fragment encompassing the critical heme-binding site was excised with simultaneous insertion of the *npt* II selection cassette conferring resistance to G418. (**b**) PCR validation of the correct integration of the construct in the *PpCYP98* genomic locus of the G418-selected mutant lines. (**c**) RT-PCR analysis of selected Δ*PpCYP98* mutants confirming the absence of *PpCYP98* transcripts. (**d**) qPCR-based evaluation of transgene copy number indicating a single integration event in the three selected mutant lines. Primer hybridization sites are indicated in (**a**). WT, wild type; M, DNA size marker (MassRuler DNA Ladder Mix, Thermo Fisher Scientific).

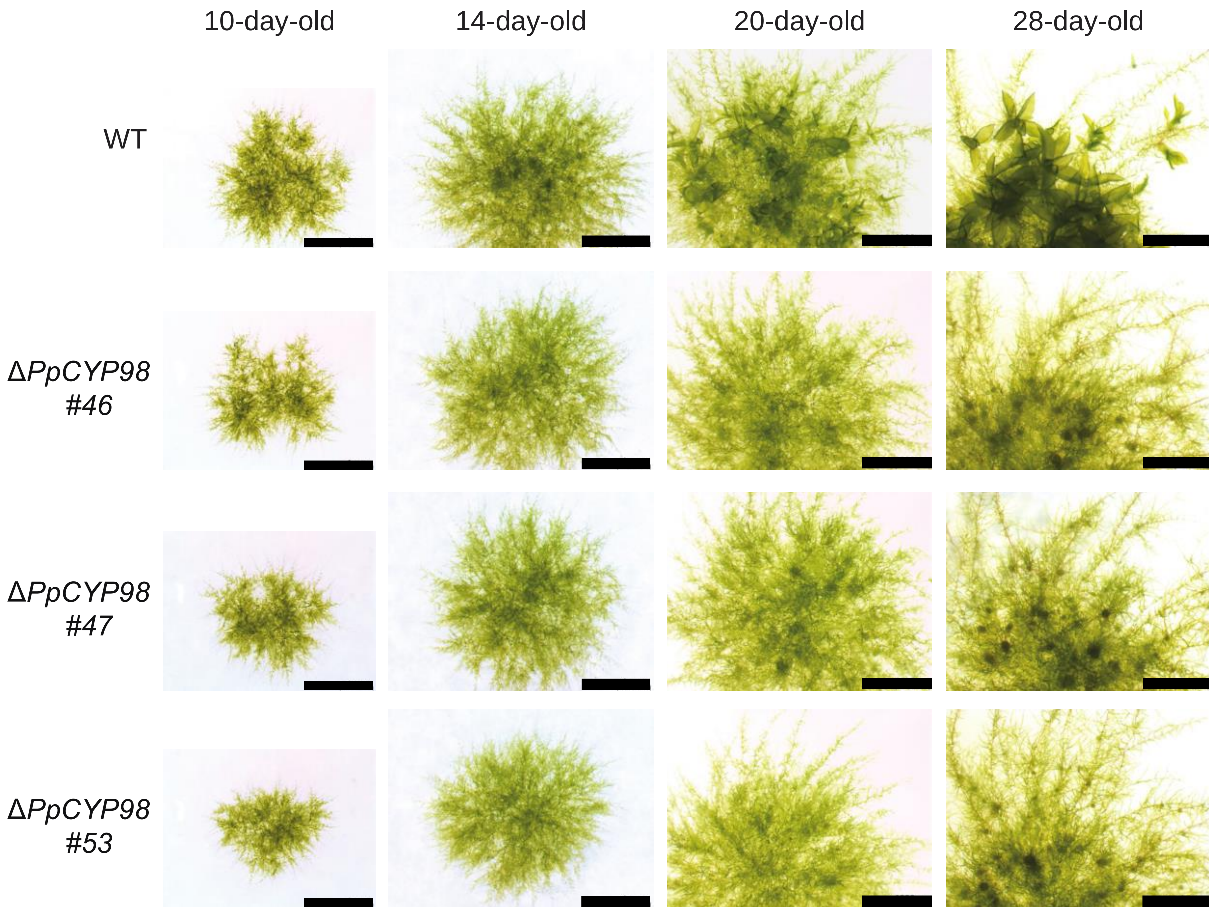

Supplementary Figure 4 | *PpCYP98* disruption consistently affects gametophore development without detrimental effect on protonema growth. Colony growth was monitored over 28 days after tissue disruption. Scale bars, 2 cm.

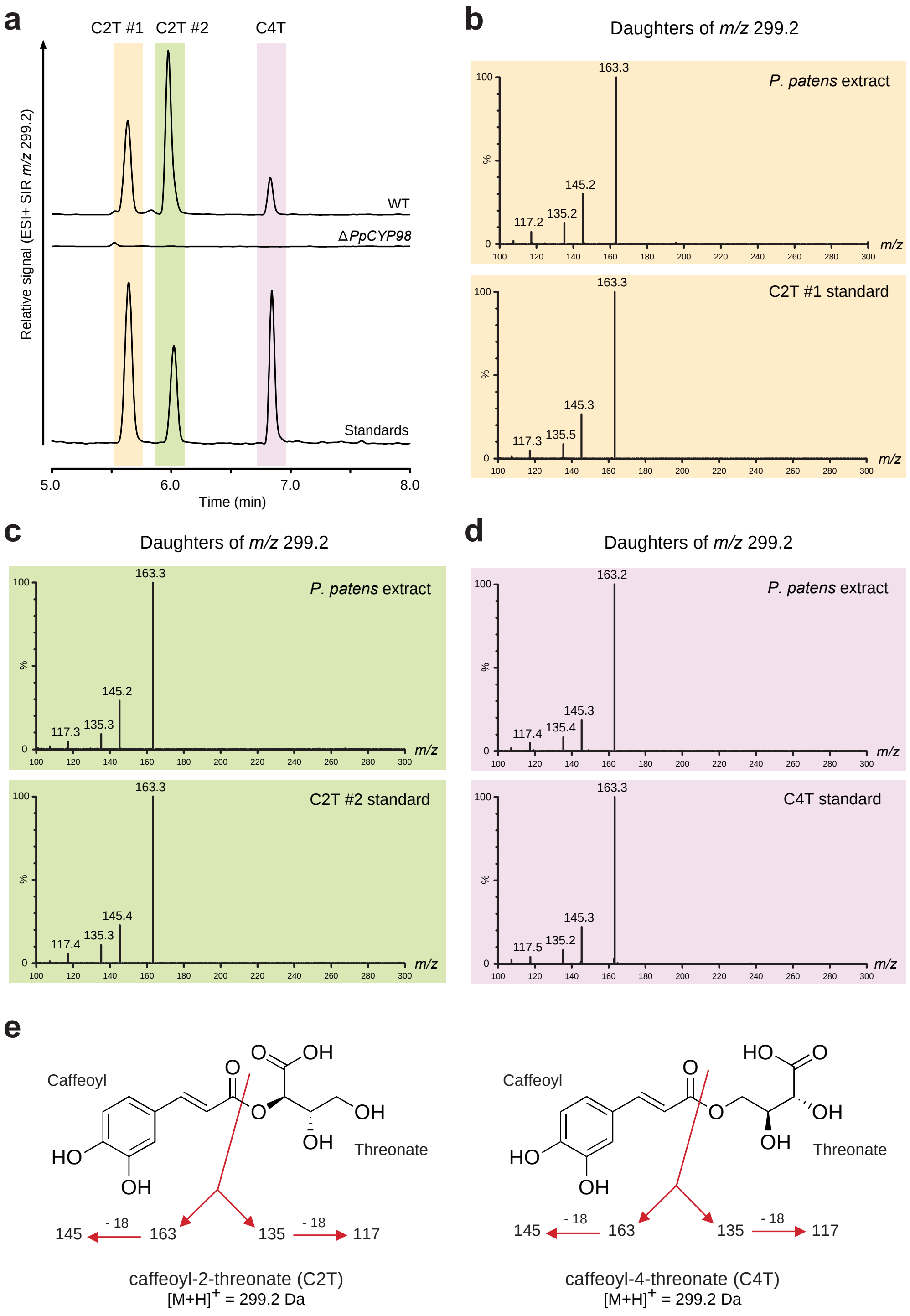

Supplementary Figure 5 | MS/MS characterization of caffeoyl-threonate esters in the plant extracts. (**a**) Selected ion recording (SIR) chromatograms of *P. patens* WT and Δ*PpCYP98* gametophore extracts show the absence of *m/z* 299.2 (ESI+) in the mutant. Ions detected in WT extract co-elute with enzymatically-generated caffeoyl-2-threonate (C2T) and caffeoyl-4-threonate (C4T) standards. Note that two caffeoyl-2-threonate isomers are present in both the *P. patens* extract and the standard. They likely result from the conversion of the two *p*-coumaroyl-2-threonate isomers also detected in the plant extract and in the standard solution (Supplementary Fig. 6). (**a-d**) Fragmentation analysis (daughter scan) of *m/z* 299.2 (ESI+) of C2T #1 (**b**), C2T #2 (**c**) and C4T (**d**) peaks in WT extracts and standards confirmed the occurrence of caffeoyl-threonate esters in *P. patens*. (**e**) Fragmentation patterns. Depicted are the forms derived from the major *p*-coumaroyl-threonate isomers present in the standard and in the moss extract.

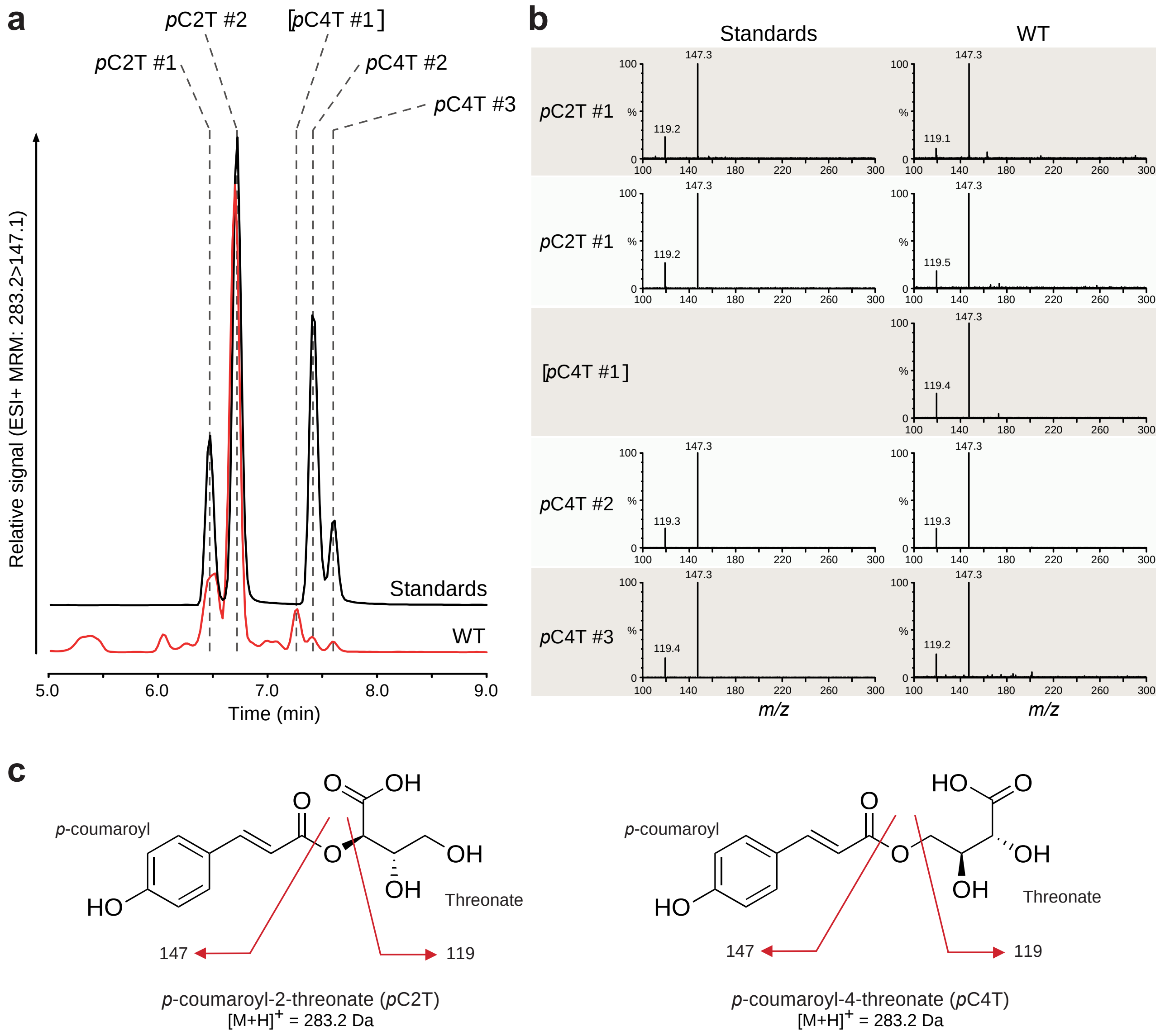

Supplementary Figure 6 | MS/MS characterization of *p*-coumaroyl-threonate esters in the plant extracts. (**a**) A multiple reaction monitoring method (ESI+ MRM: 283.2>147.1) developed using the chemically synthesized *p*-coumaroyl-threonate esters detected at least five different isomers in *P. patens* WT extract. (**b**) Fragmentation analysis (daughter scan) of *m/z* 283.2 (ESI+) confirmed their identity. Note that *p*C4T #1 does not co-elute with available standards but has a fragmentation pattern consistent with a *p*-coumaroyl-theonate structure, it is therefore indicated between brackets. The different peaks of *p*-coumaroyl-theonate are expected to correspond to different epimers, or from spontaneous conversion into *p*-coumaroyl-3-threonate. (**c**) Fragmentation patterns. Depicted are major isomers present in the synthesized standards.

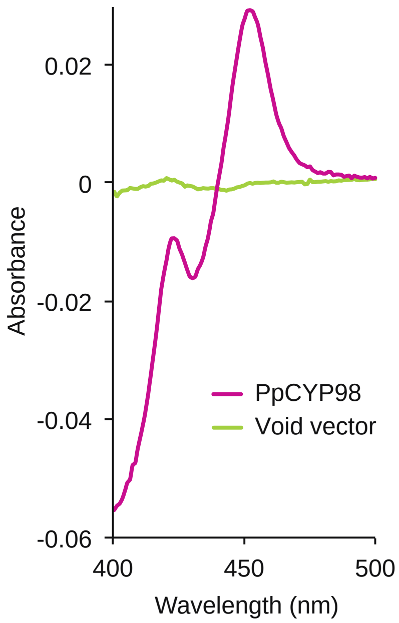

Supplementary Figure 7 | Carbon monoxide-induced UV-visible difference spectra of dithionite-reduced PpCYP98 recombinant proteins. PpCYP98 protein was expressed in the WAT11 yeast strain. Yeast microsomal fraction was used for enzyme detection and assay. Control is recorded with microsomes from yeasts transformed with the void vector.

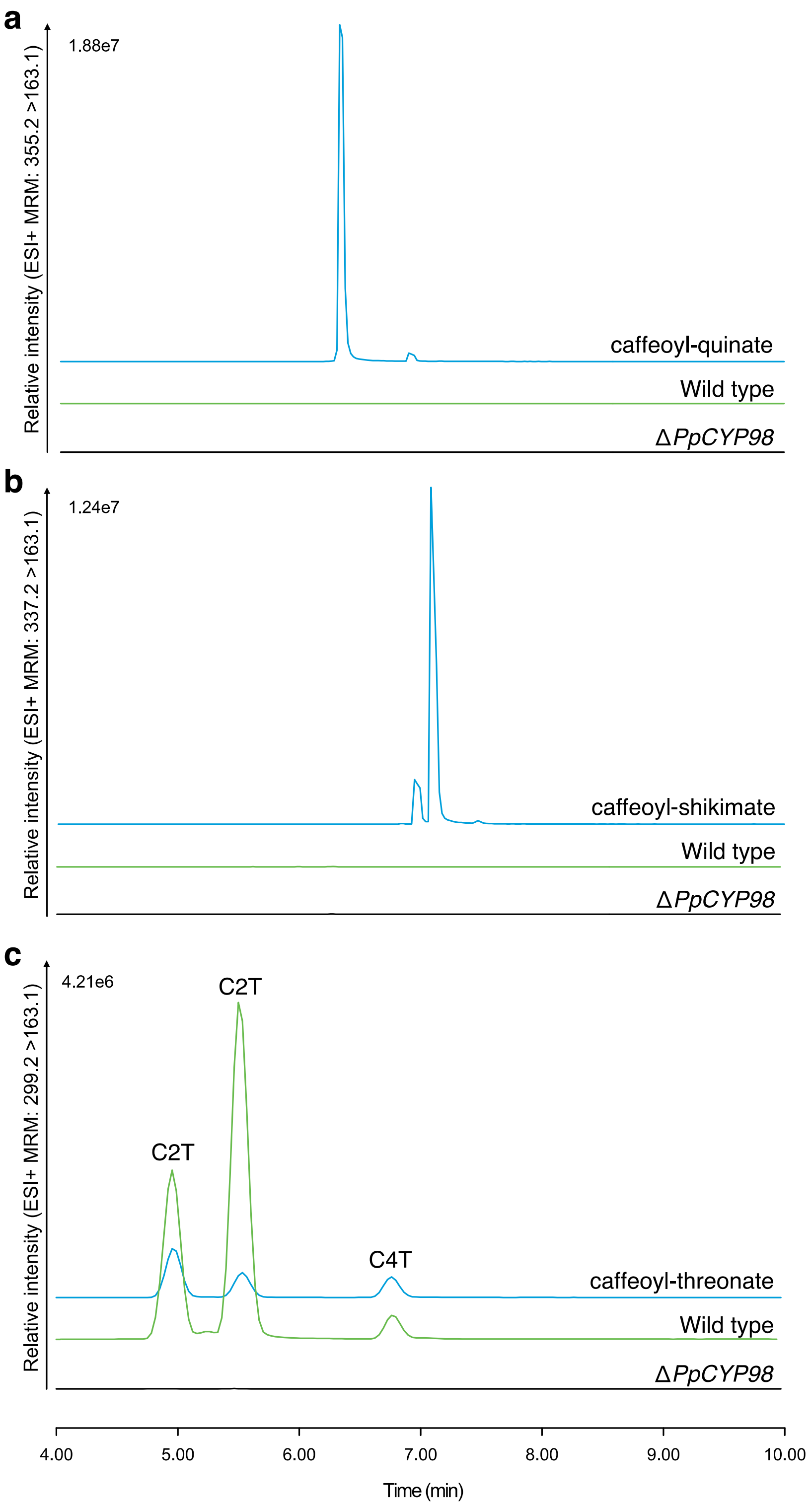

Supplementary Figure 8 | Search in *P. patens* for caffeoyl esters commonly found in angiosperms. Targeted UPLC-MS/MS analysis failed to detect caffeoyl-quinate (**a**) and caffeoyl-shikimate (**b**), but confirmed the presence of caffeoyl-threonate esters (**c**) in WT gametophore extracts. Chromatograms of standard molecules are shown as reference. Caffeoyl-quinate (i.e. chlorogenic acid) was purchased from Sigma-Aldrich; caffeoyl-shikimate and caffeoyl-threonate were produced enzymatically from their corresponding *p*-coumaroyl esters. C2T, caffeoyl-2-threonate; C4T, caffeoyl-4-threonate.

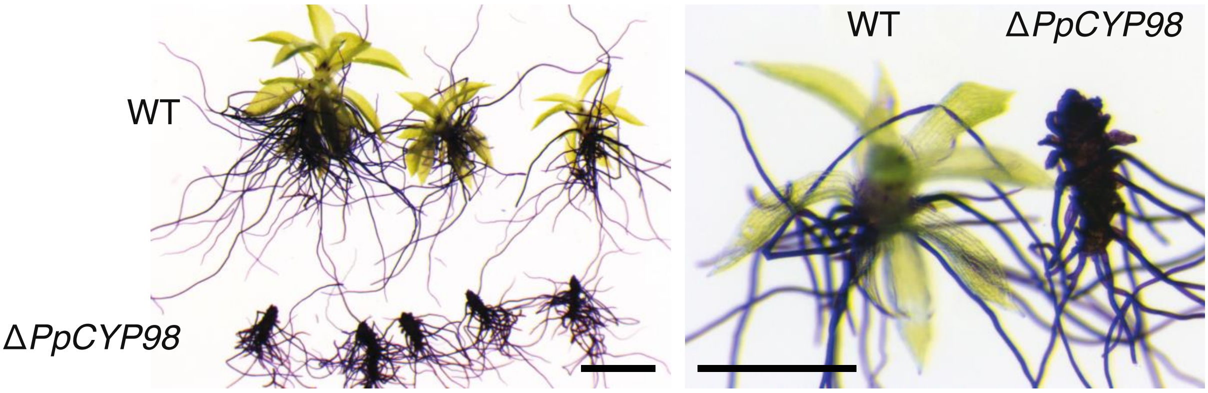

Supplementary Figure 9 | Toluidine blue permeability: close-up and additional pictures. Gametophores grown in liquid culture were immersed for two minutes in a 0.05% toluidine blue aqueous solution. They were abundantly rinsed with distilled water before pictures were recorded. Scale bars: left panel, 1 mm; right panel, 0.5 mm. Note that protonema is stained as previously reported[4].

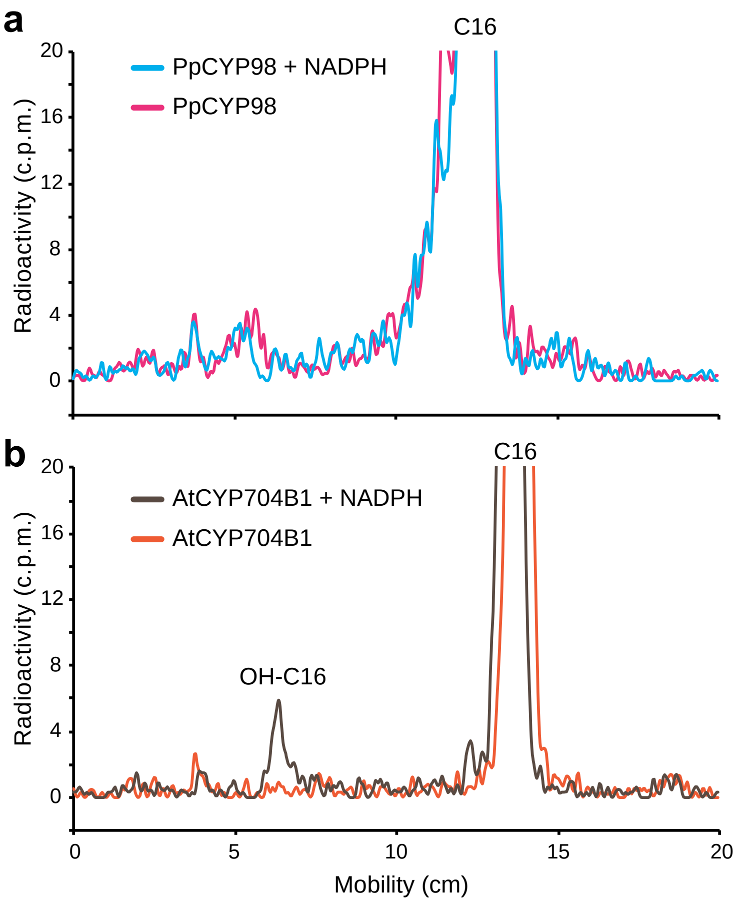

Supplementary Figure 10 | PpCYP98 does not catalyze palmitic acid hydroxylation. (**a**) The PpCYP98 recombinant protein in yeast microsomes was assayed for [1-^{14}C]palmitic acid (C16) conversion *in vitro***.** In the presence of NADPH, no oxygenated product was detected as compared with control assay without NADPH. (**b**) Assays performed with the *Arabidopsis* CYP704B1 omega-hydroxylase[5] are shown, indicating the onset of an hydroxylated C16 product.

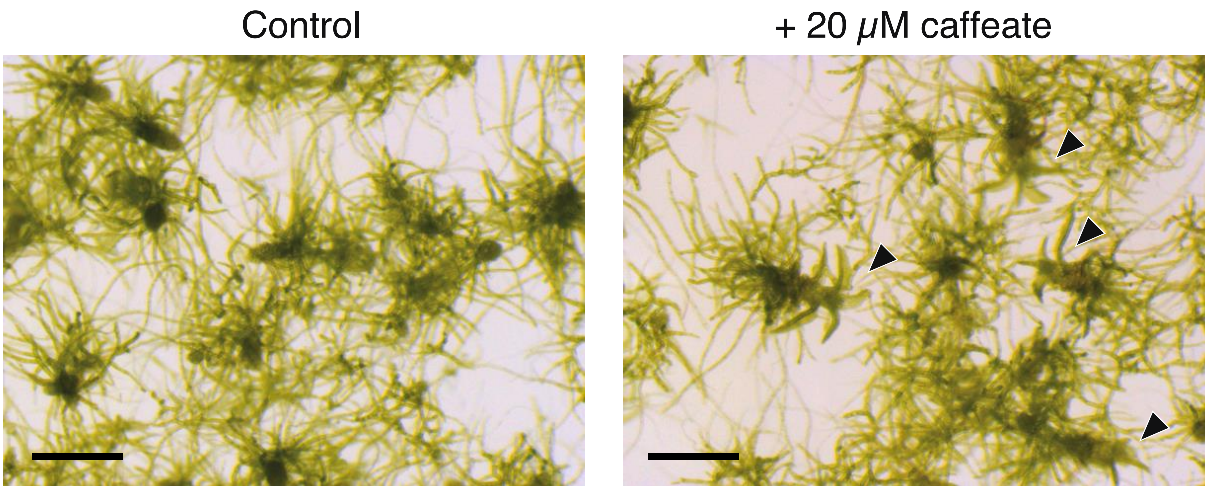

Supplementary Figure 11 | Exogenous supply of caffeate alleviates consequences of *PpCYP98* loss of function. Δ*PpCYP98* mutants were grown in liquid medium. Two weeks after the last tissue disruption, the culture was split into two subcultures, supplemented with either 20 µM caffeate or 0.05% ethanol (control). Pictures were taken 28 days after caffeic acid addition. Arrowheads point to gametophores showing growth restoration. Scale bars, 0.5 mm.

Supplementary Tables

Supplementary Table 1 | Phenolic molecules analysis in *P. patens* crude extracts.

		**Protonema**		**Gametophores**	
	RT (min)	WT	Δ*PpCYP98*	WT	Δ*PpCYP98*
Threonate esters					
Caffeoyl-2-threonate	5.60	252 (18)	n.d.	16049 (428)	n.d.
Caffeoyl-2-threonate	5.94	1210 (76)	n.d.	36651 (993)	n.d.
p-coumaroyl-2-threonate	6.33	596 (18)	659 (17)	2153 (72)	1255 (37)
p-coumaroyl-2-threonate	6.53	4220 (114)	4961 (68)	13880 (618)	9839 (258)
Caffeoyl-4-threonate	6.61	n.d.	n.d.	9265 (440)	n.d.
[caffeoyl-4-threonate]	6.81	100 (9)	n.d.	1412 (211)	n.d.
[*p*-coumaroyl-4-threonate]	7.21	710 (74)	701 (41)	1387 (68)	1559 (159)
p-coumaroyl-4-threonate	7.32	84 (13)	71 (3)	218 (51)	61 (7)
p-coumaroyl-4-threonate	7.54	43 (6)	52 (5)	258 (6)	92 (4)
Other phenolic esters					
Caffeoyl-quinate	6.29	n.d.	n.d.	n.d.	n.d.
Caffeoyl-shikimate	7.08	n.d.	n.d.	n.d.	n.d.
Free hydroxycinnamic acid					
Caffeic acid	6.58	n.d.	n.d.	n.d.	n.d.
5-OH-Ferulic acid	6.83	n.d.	n.d.	n.d.	n.d.
p-coumaric acid	7.42	n.d.	n.d.	n.d.	n.d.
Ferulic acid	7.75	n.d.	n.d.	n.d.	n.d.
Sinapic acid	7.75	n.d.	n.d.	n.d.	n.d.
Cinnamic acid	9.18	n.d.	n.d.	n.d.	n.d.

Phenolic molecules were analyzed in *P. patens* protonema and gametophores methanolic extracts using UPLC-MS/MS and MRM methods (see Supplementary Tab. 4). Results are the mean of three independent biological replicates for WT and three Δ*PpCYP98* independent mutant lines. Standard errors are indicated in brackets. The amounts of threonate phenolic esters are expressed in relative units. Molecules in brackets exhibited fragmentation patterns consistent with indicated compounds, but did not co-elute with available standards. They are thus expected to be different stereoisomers. RT, retention time; n.d., not detected.

Supplementary Table 2 | Free hydroxycinnamic acid analysis in hydrolyzed extracts.

		**Protonema**		**Gametophores**	
	RT (min)	WT	Δ*PpCYP98*	WT	Δ*PpCYP98*
Caffeic acid	6.58	0.20 (0.04)	n.d.	52.9 (5.2)	n.d.
5-OH-Ferulic acid	6.83	n.d.	n.d.	n.d.	n.d.
p-coumaric acid	7.42	1.8 (0.4)	2.3 (0.2)	25.7 (2.1)	17.0 (1.9)
Ferulic acid	7.75	n.d.	n.d.	0.56 (0.04)	n.d.
Sinapic acid	7.75	n.d.	n.d.	n.d.	n.d.
Cinnamic acid	9.18	n.d.	n.d.	n.d.	n.d.

WT and Δ*PpCYP98* crude extracts were subjected to acid hydrolysis prior to UPLC-MS/MS analysis. Results are means of three independent biological replicates for WT and three independent Δ*PpCYP98* mutant lines. Standard errors are indicated in brackets. Amounts of hydroxycinnamic acids are expressed as µmoles per gram of dry weight (µmol g^{-1} DW). RT, retention time; n.d., not detected.

Supplementary Table 3 | Primers list. Lower case letters indicate sequences involved in the cloning process, letters in red indicate restriction sites.

Log id	Name	Sequence (5' > 3')
pGEM-T linearization (GIBSON cloning)		
487	pGEM-T _F	TCTATAGTGTCACCTAAATAGCTTG
488	pGEM-T_R	GCCCTATAGTGAGTCGTATTAC
PpCYP98 disruption construct (GIBSON cloning)		
495	PpCYP98_KO_frag1_F	aatacgactcactatagggcggatccATTGTAGCAGCGCTGCTC
496	PpCYP98_KO_frag1_R	gtcatagctgCTGACTCTGCAGCCGGTG
497	NPTII_ PpCYP98_F	gcagagtcagCAGCTATGACCATGATTACGC
498	NPTII_ PpCYP98_R	gcaattggcaTTGGGTAACGCCAGGGTT
499	PpCYP98_KO_frag2_F	cgttacccaaTGCCAATTGCTTTAGAATATTT
500	PpCYP98_KO_frag2_R	tatttaggtgacactatagaggatccACTTAATTTACTCAAATTTATTTTTTATTTTTAC
PCR screening of ΔPpCYP98 transformants (direct PCR)		
589	5'F	AGCTTGTAGGGTAGAGCACA
579	5'R	TGTCGTGCTCCACCATGTTG
580	3'F	AAATCCAGTGACCTGCAGGC
590	3'R	TTGGATTCTTATTTGGTAATGATGTGA
Molecular characterization of ΔPpCYP98 mutants (RT-PCR)		
633	F1	GGCAGTCATGTGGGAGAACA
634	R1	ATGGCCCATTCCACCGAAAT
635	L21_F	GGTTGGTCATGGGTTGCG
636	L21_R	GAGGTCAACTGTCTCGCC
Evaluation of ΔPpCYP98 mutants transgene copy number (qPCR)		
719	5'qF	TGGTGGCAGCTTTGTTCAAG
720	5'qR	ACACAATGCGGGTGATGTTG
721	3'qF	TGCACAAAGATACCCAACGG
722	3'qR	GCAGCGCCAAAACTTTTCAAG
669	PpCLF_5915_qF	AGCAATGTCCGTGCCTACTT
670	PpCLF_5981_qF	TTGTAAGAATCACTCACCCACAG
671	PpCLF_7739_qF	GTATTGGCGATCCCACTCTT

672	PpCLF_7804_qF	GCATAAAATAGGTCACAGATTGAGG
PpCYP98:uidA construct (GIBSON cloning)		
609	PpCYP98_GUS_frag1_F	aatacgactcactatagggcgaattcAGTATGATTTGAGCGAGACCAC
610	PpCYP98_GUS_frag1_R	ttaagcctgcCGAAGGGGATGATCCGTT
611	GUS_ PpCYP98_F	atccccttcgGCAGGCTTAATGTTACGTC
612	GUS_ PpCYP98_R	agacgctccaTCATTGTTTGCCTCCCTGCTG
613	PpCYP98_GUS_frag2_F	caaacaatgaTGGAGCGTCTGCACTCGT
614	PpCYP98_GUS_frag2_R	tatttaggtgacactatagagaattcGGTTGTTTTGGTAAAAGCCTAGG
PCR screening of PpCYP98:uidA transformants (direct PCR)		
802	5'GUS	CGGCTGCAGAGTCAGAAGAA
803	uidA5'	TCCACAGTTTTCGCGATCCA
808	uidA3'	CGTCGTCGGTGAACAGGTAT
807	3'GUS	ACCAGATCTGCAACCAATGA

Supplementary Table 4 | UPLC-MS/MS multiple reaction monitoring (MRM) methods.

Molecule	MW	Ionization mode	Cone voltage	Collision energy	MRM transition
Cinnamic acid	148.16	ESI+	18	16	149.2 > 103.1
p-coumaric acid	164.16	ESI+	22	10	165.2 > 147.1
Caffeic acid	180.16	ESI+	20	12	181.2 > 163.1
Ferulic acid	194.18	ESI+	20	8	195.3 > 177.2
5-OH-Ferulic acid	210.18	ESI+	20	6	211.3 > 193.1
Sinapic acid	224.21	ESI+	20	8	225.4 > 207.2
p-coumaroyl-threonate	282.07	ESI+	14	14	283.2 > 147.1
Caffeoyl-threonate	298.07	ESI+	14	14	299.2 > 163.1
Caffeoyl-quinate	354.31	ESI+	17	16	355.2 > 163.1
Caffeoyl-shikimate	336.08	ESI+	17	16	337.2 > 163.1

Supplementary Note 1

Yeast-optimized PpCYP98 sequence:

ATGGCCGTTATGTGGGAAAACACCTATACCGTCGCTGCTATCGTTGCCGCCTTATTATTCATGATGTACAAATCCTT
GAGAAGTTCACATAAATTGCCACCTGGTCCTAGACCATTGCCTGTTGTTGGTAATTTGACTCACATTACACCAGTTA
GATTCAAATGTTTCATGGAATGGGCTCAAACATACGGTTCCGTCTTGAGTGTTTGGATGGGTCCTACCTTAAACGTC
GTTGTATCTTCAGCCGATGCTGCAAAAGAAATGTTGAAGGAAAGAGACCATGCTTTGTCCAGTAGACCATTAACAAG
AGCCGCTGCAAGATTTTCCAGAAATGGTCAAGATTTGATTTGGGCAGACTATGGTCCTCACTACGTAAAAGTCAGAA
AGGTTTGTACCTTGGAATTGTTTACTTTTAAAAGATTGGAAAGTTTAAAGCCAGTTAGAGAAGATGAAGTAGGTGCT
ATGGTCGCCGCTTTGTTTAAAGATTGCGCAGATTCAAGACCTTTGAATTTGAAGAAATACGTTTCAGCAATGGCCTT
CAATAACATCACTAGAATCGTTTTCGGTAAAAGATTCGTAGATGACAAGGGTAATATCGATAACCAAGGTGTCGAGT
TTAAAGAAATAGTTTCTCAGGGTATGAAATTGGGTGCTTCTTTAAAGATGTCAGAACATATCCCATACTTGAGATGG
ATGTTCCCTTTGCAAGAAGAAGAATTTGCAAAGCATGGTGCCAGAAGAGATAATTTGACAAAGGCTATAATGCAAGA
ACACAGATTACAATCTCAAAAGAATGGTCCAGGTCATCACTTCGTTGATGCATTGTTATCCATGCAAAAGCAATACG
ACTTAAGTGAAACTACAATCATCGGTTTGTTGTGGGATATGATTACTGCTGGTATGGACACCACTGCAATTTCTGTT
GAATGGGCTATAGCAGAATTGGTTAGAAATCCAGATGTTCAAGTAAAAGCTCAACAAGAATTAGATCAAGTCGTTGG
TCAAGACAGAGTAGTCACCGAAGCAGATTTTTCACAATTGCCATATTTGCAAGCCGTTGCTAAAGAAGCCTTGAGAT
TACATCCACCTACTCCATTGATGTTACCTCACAAAGCAACAGAAACCGTAAAGATAGGTGGTTATGATGTCCCAAAG
GGTACTGTTGTACATTGTAATGTCTACGCTATCTCAAGAGACCCTACAGTTTGGGAAGAACCATTGAGATTCAGACC
TGAAAGATTCTTAGAAGAAGATATTGACATTAAGGGTCATGATTACAGATTGTTACCATTTGGTGCCGGTAGAAGAG
TATGCCCTGGTGCTCAATTGGGTTTAAACATGGTTCAATTGATGTTAGCAAGATTGTTACATCACTTTTCCTGGGCC
CCACCTCCAGGTGTTACACCAGCAGCCATTGATATGACCGAAAGACCTGGTGTCGTTACTTTCATGGCTGCACCATT
GCAAGTTTTAGCTACACCTAGATTGAGAGCCGCTTTATATAAAAATGGTTCTTCACCATCATAA

Supplementary Note 2

Chemical synthesis of p-coumaroyl-threonate esters

All reactions were carried out under an argon atmosphere. Chemicals and solvents were purchased from Sigma-Aldrich and were used without further purification. Analytical TLC were performed using silica gel plates Merck 60F254 and plates were visualized by exposure to ultraviolet light. Compounds were purified on silica gel Merck 60 (particle size 0.040-0.063nm) or using Armen spot flash chromatography (reverse phase column: AIT 50g C18). Yields refer to isolated compounds, estimated to be >97% pure as determined by ^{1}H NMR or HPLC. ^{1}H and ^{13}C NMR spectra were recorded on Bruker Avance Spectrometer operating at 400 MHz and 100 MHz, respectively. All chemical shift values δ and coupling constants *J* are quoted in ppm and in Hz, respectively, multiplicity (s= singulet, d= doublet, t= triplet, q= quartet, quin = quintet, m= multiplet, br= broad). Analytical RP-HPLC-MS was performed using a LC-MSD 1200SL Agilent with a Thermo Hypersilgold® column (C18, 30 mm x 1 mm; 1.9 µm) using the following parameters: 1) The solvent system: A (acetonitrile) and B (0.05% TFA in H_2O); 2) A linear gradient: t= 0 min, 98% B; t = 5 min, 5% B; t = 6 min, 5% B; t = 7 min, 98% B; t = 9 min, 98% B; 3) Flow rate of 0.3 $mL.min^{-1}$; 4) Column temperature: 50°C; 5) The ratio of products was determinate by integration of spectra recorded at 210 nm or 254 nm; 6) Ionization mode : MM-ES+APCI. HPLC were performed using a Dionex UltiMate 300 using the following parameters: Flow rate of 0.5 mL/min, column temperature: 30°C, solvent system: A (MeOH) and B (0.05% of THA in H_2O), t = 0 min to 1 min: 50 to 60% of B then t = 1 min to t = 10 min: 60 to 100% of B and t = 10 min to t = 15 min: 100% of B. Melting points were realized using a Büchi Melting point B-540.

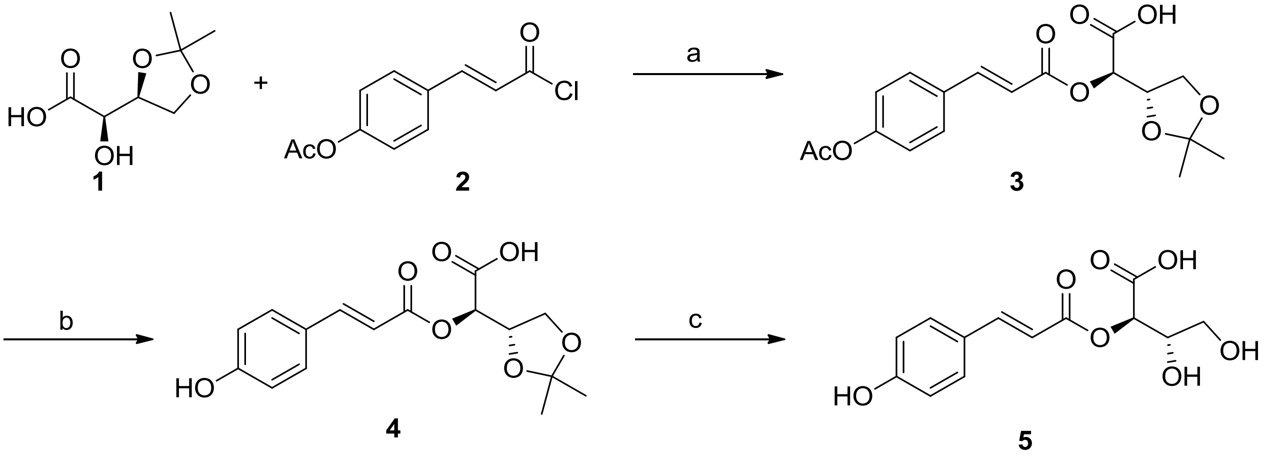

Reaction conditions: a. DCE, pyridine, DMAP, 55 °C, 4h; b. MeOH/H_2O (8:2), NH_4OH, 25 °C, 60h; c. TFA/H_2O (1:1), DCM, 0-25 °C, 6h, 12% global yield (3 steps).

(R)-2-((S)-2,2-dimethyl-1,3-dioxolan-4-yl)-2-hydroxyacetic acid (1)

From Bueno *et al.*[6]

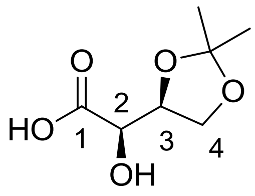

To a suspension of calcium 3,4-*O*-isopropylidene-L-threonate (1.00 g, 2.56 mmol) in water (15 mL), cooled in an ice-water bath, was added HCl (2M) up to pH 3-4. The solution was extracted with EtOAc (9 x 25 mL) and the pH of the aqueous solution was checked from time to time in order to keep it at about 3. The organic phase was dried over anhydrous Na_2SO_4 and concentrated under reduced pressure to obtain 3,4-*O*-isopropylidene-L-threonic acid as a white solid (600 mg, 66%). mp 71-73 ºC

^{1}H NMR (400 MHz, $CDCl_3$): δ 1.37 (s, 3H, CH_3), 1.45 (s, 3H, CH_3), 4.05 (dd, 1H, J = 6.8, 8.4 Hz, H_{4b}), 4.14 (dd, 1H, J = 6.8, 8.8 Hz, H_{4a}), 4.20 (d, 1H, J = 3.2 Hz, H_2), 4.46 (ddd, 1H, $J_{2,3}$ = 3.2, $J_{3,4a}$ = $J_{3,4b}$ = 6.8 Hz, H_3).

^{13}C NMR (100 MHz, $CDCl_3$): δ 25.3, 26.2 (C$\underline{(CH_3)_2}$), 65.8 (C_4), 70.3 (C_2), 76.2 (C_3), 110.4 ($\underline{C}(CH_3)_2$), 175.6 (C_1).

4-(acetyloxy)-(E)-cinnamoyl chloride (2)

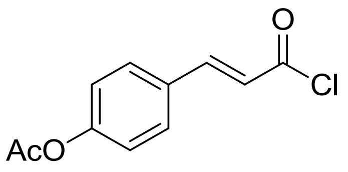

Oxalyl chloride (615 mg, 0.42 mL, 4.85 mmol, 2.0 equiv) was slowly added to a stirred mixture of the (*E*)-3-(4-acetoxyphenyl)-2-propenoic acid (500 mg, 2.4 mmol, 1.0 equiv) and N,N-dimethylformamide (2-3drops) in dry CH_2Cl_2 (5 mL) at 0 °C under an argon atmosphere. The reaction mixture was stirred at room temperature for an additional 2 h and evaporated to dryness with the help of cyclohexane to give the corresponding acid chloride in a quantitative yield. This acid chloride was used directly in the next step without further purification.

(R)-2-(((E)-3-(4-acetoxyphenyl)acryloyl)oxy)-2-((S)-2,2-dimethyl-1,3-dioxolan-4-yl)acetic acid (3)

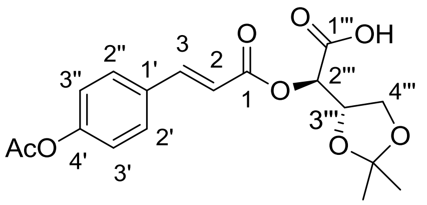

A solution of 4-acetyl coumaric chloride (300 mg, 1.3 mmol, 1.1 equiv) in dichloroethane (5 mL) was slowly added to a stirred solution of 4-isopropylidene-L-threonic acid (214 mg, 1.2 mmol, 1.0 equiv), pyridine (0.245 mL, 3.0 mmol, 2.5 equiv) and DMAP (44.5 mg, 0.36 mmol, 0.3 equiv) in dichloroethane (10 mL). The resulting solution was then stirred for 4h at 55 °C. After cooling to room temperature, the reaction mixture was treated successively with saturated aqueous solution of NH_4Cl (5 mL), saturated aqueous solution of $NaHCO_3$ (10 mL) and H_2O (10mL) and dried over anhydrous Na_2SO_4. The solvent was removed under reduced pressure and crude 3,4-O-isopropylidene-L-threonic acid derivative was used for the next step without further purification.

^{1}H NMR (400 MHz, CD_3OD): δ 1.36 (s, 3H, CH_3), 1.46 (s, 3H, CH_3), 2.29 (s, 3H, CH_3), 4.0 (dd, 1H, J = 6.4, 8.8 Hz, $H_{4'''}$), 4.13 (dd, 1H, J = 6.8, 8.8 Hz, $H_{4'''}$), 4.60 (dd, 1H, J = 5.6, 6.4 Hz, $H_{3'''}$), 5.12 (d, 1H, J = 5.2 Hz, $H_{2'''}$), 6.60 (d, 1H, J = 16 Hz, H_2), 7.15-7.18 (m, 2H, $H_{3'/3''}$), 7.65-7.68 (m, 2H, $H_{2'/2''}$), 7.78 (d, 1H, J = 16 Hz, H_3).

(R)-2-((S)-2,2-dimethyl-1,3-dioxolan-4-yl)-2-(((E)-3-(4-hydroxyphenyl)acryloyl)oxy)acetic acid (4)

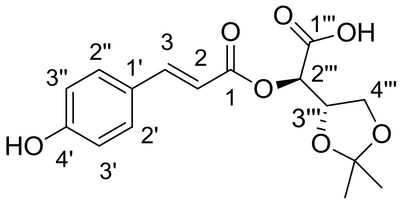

To a solution of derivative **3** (440 mg, 1.2 mmol, 1.0 equiv) in a mixture of MeOH/H_2O (8:2, 10 mL) was added NH_4OAc (745 mg, 9.7 mmol, 8.0 equiv). The resulting solution was stirred at room temperature for 60 h. The solvent was removed under reduced pressure and the crude mixture was extracted with EtOAc (3 x 10 mL). The organic phase was dried over anhydrous Na_2SO_4 and concentrated under reduced pressure and the crude mixture was purified by reversed-phase flash chromatography to afford 1:1 mixture (110 mg) of desired product **4**, , and diol compound **5** by the further deprotection of 3,4-O-isopropylidene group.

^{1}H NMR (400 MHz, CD_3OD): δ 1.36 (s, 3H, CH_3, **4**), 1.47 (s, 3H, CH_3, **4**), 3.61-3.63 (m, 2H, $H_{4'''}$, **5**), 3.98 (dd, 1H, J = 6.0, 8.8 Hz, $H_{4'''}$, **4**), 4.15 (dd, 1H, J = 6.8, 8.8 Hz, $H_{4'''}$, **4**), 4.19 (ddd, 1H, $J_{2''',3'''}$ = 2.4, $J_{3''',4a'''}$ = $J_{3''',4b'''}$ = 6.4 Hz, $H_{3'''}$, **5**), 4.63 (ddd, 1H, $J_{2''',3'''}$ = 4.4, $J_{3''',4a'''}$ = $J_{3''',4b'''}$ = 5.6 Hz, $H_{3'''}$, **4**), 5.16 (d, 1H, J = 4.8 Hz, $H_{2'''}$, **4**), 5.27 (d, 1H, J = 2.4 Hz, $H_{2'''}$, **5**), 6.40, 6.44 (2d, 2H, J = 16 Hz, H_2, **4**, **5**), 6.80-6.84 (m, 4H, $H_{3'/3''}$, **4**, **5**), 7.46-7.51 (m, 4H, $H_{2'/2'}$, **4**, **5**·), 7.74 (t, 2H, J = 16 Hz, H_3, **4**, **5**)

(2R,3S)-3,4-dihydroxy-2-(((E)-3-(4-hydroxyphenyl)acryloyl)oxy)butanoic acid (5)

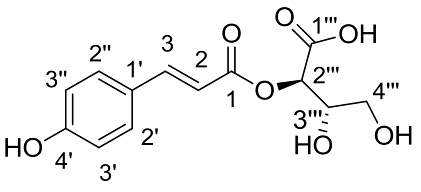

A mixture of TFA/H_2O (1:1, 3,2 mL) was slowly added to a solution of the mixture products (**4**, **5,** 110 mg), obtained in the early step, in CH_2Cl_2 (2 mL), cooled in an ice-water bath. The resulting solution was stirred at room temperature for 6 h. The reaction mixture was evaporated to dryness with help of methanol. The crude was purified by reversed-phase flash chromatography to afford **5** as a white solid (40 mg, 12% (3 steps)). mp = 160-162 °C

^{1}H NMR (400 MHz, CD_3OD): δ 3.58-3.66 (m, 2H, $H_{4'''}$), 4.18-4.21 (m, 1H, $H_{3'''}$), 5.26 (d, 1H, J = 2.0 Hz, $H_{2'''}$), 6.44 (d, 1H, J = 15.6 Hz, H_2), 6.80-6.84 (m, 2H, $H_{3'/3''}$), 7.47-7.51 (m, 2H, $H_{2'/2''}$), 7.75 (d, 1H, J = 16 Hz, H_3).

^{13}C NMR (100 MHz, CD_3OD): δ 63.2 ($C_{4'''}$), 72.7 ($C_{2'''}$, $C_{3'''}$), 114.6 (C_2), 116.8 ($C_{3'/3''}$), 127.2 ($C_{1'}$), 131.3 ($C_{2'/2''}$), 147.4 (C_3), 161.4 (C_1), 168.5 ($C_{1'''}$).

HRMS (M+Na$^+$): 305.0636 (calcd for $C_{13}H_{14}NaO_7$ 305.0637).

(2R,3S)-2,3-dihydroxy-4-(((E)-3-(4-hydroxyphenyl)acryloyl)oxy)butanoic acid (6)

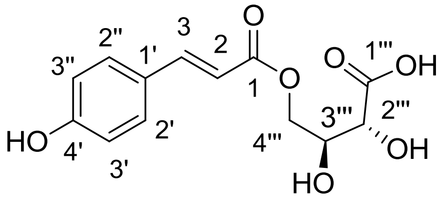

Compound **5** (5 mg) was dissolved in phosphate buffer (0.1 M, pH 7.4) and the resulting solution was stirred at 80 °C for 1 h. The reaction mixture was directly purified by reversed-phase flash chromatography to afford **6** as a white solid (2.7 mg, 54%).

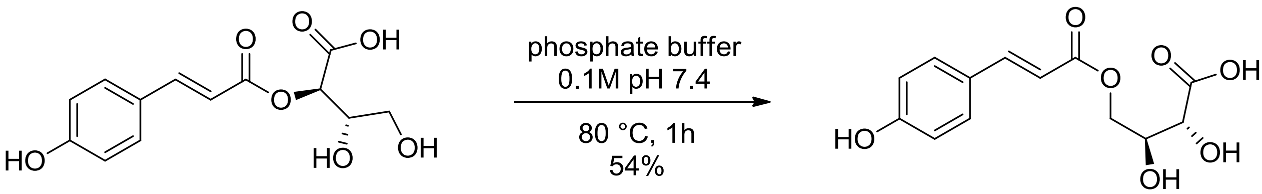

5 **6**

^{1}H NMR (400 MHz, CD_3OD): δ 3.96 (d, 1H, J = 2.0 Hz, $H_{2'''}$), 4.16-4.20 (m, 1H, $H_{3'''}$), 4.24-4.26 (m, 2H, $H_{4'''}$), 6.20 (d, 1H, J = 15.6 Hz, H_2), 6.65 (d, 2H, J = 8.8 Hz, $H_{3'/3''}$), 7.35 (d, 2H, J = 8.8 Hz, $H_{2'/2''}$), 7.63 (d, 1H, J = 15.6 Hz, H_3).

^{13}C NMR (100 MHz, CD_3OD): δ 66.6 ($C_{4'''}$), 71.9 ($C_{3'''}$), 73.4 ($C_{2'''}$), 111.5 (C_2), 119.4 ($C_{3'/3''}$), 131.5 ($C_{2'/2''}$), 148.0 (C_3), 169.9 (C_1), 178.9 ($C_{1'''}$).

HRMS ($M+Na^+$): 305.0629 (calcd for $C_{13}H_{14}NaO_7$ 305.0637).

Supplementary References

1. Ortiz-Ramirez C*, et al.* A transcriptome atlas of *Physcomitrella patens* provides insights into the evolution and development of land plants. *Mol Plant* **9**, 205-220 (2016).
2. Winter D, Vinegar B, Nahal H, Ammar R, Wilson GV, Provart NJ. An "Electronic Fluorescent Pictograph" browser for exploring and analyzing large-scale biological data sets. *PLoS One* **2**, e718 (2007).
3. Landberg K*, et al.* The moss *Physcomitrella patens* reproductive organ development is highly organized, affected by the two *SHI/STY* genes and by the level of active auxin in the *SHI/STY* expression domain. *Plant Physiol* **162**, 1406-1419 (2013).
4. Wyatt HD, Ashton NW, Dahms TE. Cell wall architecture of *Physcomitrella patens* is revealed by atomic force microscopy. *Botany* **86**, 385-397 (2008).
5. Dobritsa AA*, et al.* CYP704B1 is a long-chain fatty acid omega-hydroxylase essential for sporopollenin synthesis in pollen of Arabidopsis. *Plant Physiol* **151**, 574-589 (2009).
6. Bueno M, Molina I, Galbis JA. 1,4-Dioxane-2,5-dione-type monomers derived from l-ascorbic and d-isoascorbic acids. Synthesis and polymerisation. *Carbohydr Res* **344**, 2100-2104 (2009).
